# *SF3B1* mutation accelerates the development of CLL via activation of the mTOR pathway

**DOI:** 10.1172/jci.insight.184280

**Published:** 2025-07-22

**Authors:** Bo Zhang, Prajish Iyer, Meiling Jin, Elisa Ten Hacken, Zachary J. Cartun, Kevyn L. Hart, Mike Fernandez, Kristen Stevenson, Laura Rassenti, Emanuela M. Ghia, Thomas J. Kipps, Donna Neuberg, Ruben Carrasco, Wing C. Chan, Joo Y. Song, Yu Hu, Catherine J. Wu, Lili Wang

**Affiliations:** 1 Department of Systems Biology, Beckman Research Institute, City of Hope Comprehensive Cancer Center, Monrovia, California, USA.; 2 Institute of Hematology, Union Hospital, Tongji Medical College, Huazhong University of Science and Technology, Wuhan, China.; 3 Department of Medical Oncology and; 4 Department of Data Science, Dana-Farber Cancer Institute, Boston, Massachusetts, USA.; 5 Moores Cancer Center, University of California San Diego Health, La Jolla, California, USA.; 6 Department of Hematology & Hematopoietic Cell Transplantation and; 7 Toni Stephenson Lymphoma Center, Beckman Research Institute, City of Hope Comprehensive Cancer Center, Duarte, California, USA.

**Keywords:** Hematology, Oncology, Leukemias

## Abstract

RNA splicing factor *SF3B1* is one of the most recurrently mutated genes in chronic lymphocytic leukemia (CLL) and frequently co-occurs with chromosome 13q deletion [*del*(13q)]. This combination is associated with poor prognosis in CLL, suggesting these lesions increase CLL aggressiveness. While *del*(13q) in murine B cells (minimal deleted region of 13q14 includes DLEU1, DLEU2, and miR15a-16-1; *Mdr* mice), but not expression of *Sf3b1*-K700E, drives the initiation of CLL, we hypothesize that *SF3B1* mutation accelerates CLL progression. In this study, we crossed mice with a B cell–specific *Sf3b1*-K700E allele with *Mdr* mice to determine the impact of *Sf3b1* mutation on CLL progression. We found that the co-occurrence of these 2 lesions in murine B cells caused acceleration of CLL. We showed that *Sf3b1*-K700E impacted alternative RNA splicing of nuclear factor of activated T cells C1 (*Nfatc1*) and activated mTOR signaling and the MYC pathway, contributing to CLL acceleration. Moreover, concurrent inhibition of RNA splicing and the mTOR pathway led to cell death in vitro and in vivo in murine CLL cells with *SF3B1* mutation and *del*(13q). Our results thus suggest that *SF3B1* mutation contributes to the aggressiveness of CLL by activating the mTOR pathway through alternative splicing of *Nfatc1*, providing a rationale for targeting mTOR and RNA splicing in the subset of CLL patients with both *SF3B1* mutations and *del*(13q).

## Abstract 2

Mutant *SF3B1* activates the mTOR pathway to contribute to aggressive CLL

## Introduction

RNA splicing factor *SF3B1* is recurrently mutated in various cancer types, including chronic lymphocytic leukemia (CLL) (1–3), myeloid dysplasia (MDS) (4, 5), acute myeloid leukemia (6), uveal melanoma (UVM) (7), and breast and pancreatic cancers (8–10). Over the past few years, murine models based on the tissue-specific expression of *Sf3b1*-K700E have revealed that aberrant splicing events drive progression in these cancers (11–16). Specifically, in breast cancer, *SF3B1* mutations induce a recurrent pattern of aberrant splicing, leading to activation of AKT and NF-κB, enhanced cell migration, and tumorigenesis in mammary epithelial and breast cancer cells (11). In UVM, *SF3B1* mutation drives aberrant RNA splicing of BRD9, a key component essential for the noncanonical BRG1/BRM-associated factor chromatin-remodeling complex, which is required to maintain *SF3B1*-mutant cancers (12). Together, these results suggest that *SF3B1* mutation uses aberrant RNA splicing to promote tumorigenesis via regulation of tumor-specific pathways, serving as a unique vulnerability in cancers with these mutations.

CLL is characterized by CD19^+^CD5^+^ B cells accumulating in blood, bone marrow, lymph nodes, and spleen (17). *SF3B1* mutations occur in over 20% of CLL samples, often co-occurring with chromosome 13q deletion [*del*(13q)] or 11q deletion [*del*(11q))] (18, 19). More than 50% of *SF3B1* mutations localize at the K700 site (2). This gene mutation tends to be subclonal in CLL, and its presence is associated with a shorter time to first therapy (20), suggesting an essential role in driving the aggressiveness of CLL. We previously generated a murine model, which confirmed that mutated *SF3B1* in conjunction with ataxia-telangiectasia-mutated (*ATM*) deletion caused the onset of low penetrance CLL by overriding cellular senescence imposed by *SF3B1* mutation (21). However, whether the function of this mutation is to accelerate CLL remains elusive; if so, the underlying mechanisms contributing to such disease acceleration are yet to be elucidated.

Here, we used an existing *Mdr*-deleted CLL murine model [mimicking clonal *del*(13q)] (22) and crossed this line with mice expressing a conditional *Sf3b1*-K700E allele to allow the coexpression of *Sf3b1* mutation and *del*(13q) in murine B cells. With these mice, we investigate how *Sf3b1* mutation impacts oncogenic pathways to contribute to CLL acceleration via an RNA splicing–dependent mechanism.

## Results

### Coexpression of Sf3b1-K700E with del(13q) in B cells accelerates the onset of CLL in vivo.

**Figure 1:**
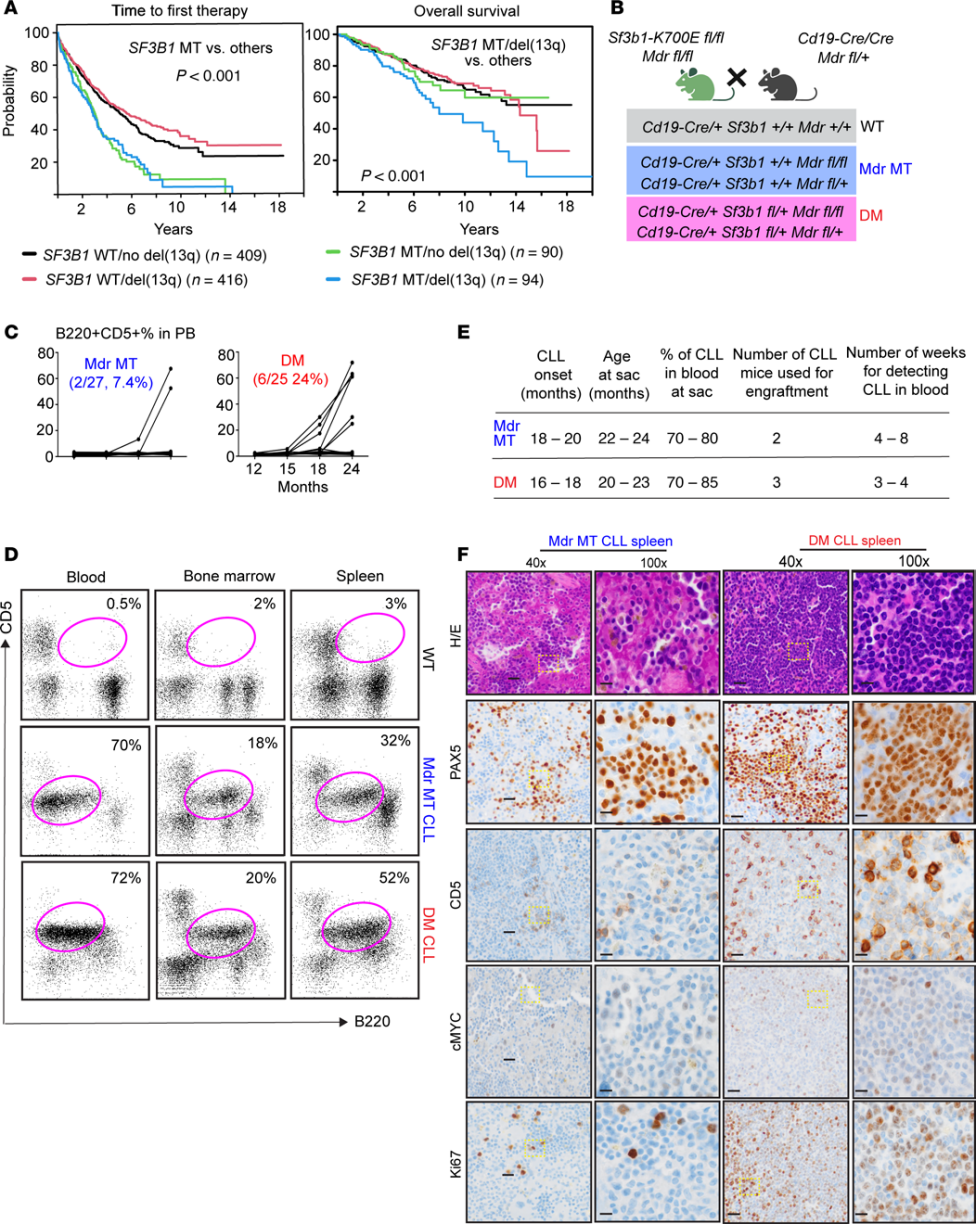
Coexpression of *Sf3b1-*K700E and *del*(13q) in murine B cells leads to aggressive CLL. (**A**) *SF3B1* mutations and *del*(13q) are associated with shorter time to therapy and inferior overall survival in CLL. Log-rank test, *P* < 0.001. MT, mutant; WT, wild-type. (**B**) Mouse crossing strategy and genotype used in the current study. (**C**) The change curve of CLL-like cell (B220^+^CD5^+^) percentage within the lymphocyte population from peripheral blood cells from *Mdr* MT and DM mouse groups. *X* axis time units are 12, 15, 18, and 24 months. *Y* axis units are %B220^+^CD5^+^ cells. (**D**) Flow cytometry data identified B220^+^CD5^+^ cells in blood, spleen, and bone marrow derived from WT, *Mdr* mutant CLL, and DM mutant CLL mice (**E**) Summary of *Mdr* MT and DM cohort and engraftment in NSG mice. Table showing (left to right): time to CLL onset (months), age at sacrifice (months), percentage of circulating B220^+^CD5^+^ cells at sacrifice, number of donor mice used for transplantation, and time to first detection of circulating B220^+^CD5^+^ cells in NSG recipients (weeks). Detection in recipients was defined as >5% B220^+^CD5^+^ cells in PB by flow cytometry. (**F**) Immunohistochemical staining of PAX5, CD5, MYC, and Ki67 along with H&E on sections of spleen derived from *Mdr* MT CLL and DM CLL. Black scale bars on 40× and 100× indicate 20 mm and 10 mm, respectively.

A recent comprehensive survey of CLL genomics data from more than 1,000 patients (https://cllmap.org/) confirmed *SF3B1* as one of the most recurrently mutated genes in CLL (184 of 1,009, 18.2%) (23). Remarkably, 51% of *SF3B1* mutations co-occurred with *del*(13q), one of the most common chromosomal abnormalities in CLL (23). *SF3B1* mutations were associated with a significantly shorter time to first therapy, independent of *del*(13q) status: *SF3B1* mut/*del*(13q) versus *SF3B1* wt/no *del*, *P* < 0.0001; *SF3B1* mut/*del*(13q) versus *SF3B1* wt/*del*(13q), *P* < 0.0001; *SF3B1* mut/*del*(13q) versus *SF3B1* mut/no *del*(13q), *P* = 0.89 (Figure 1A). However, the co-occurrence of *SF3B1* mutation and *del*(13q) was associated with significantly inferior overall survival compared with patients with *SF3B1* mutation alone, *del*(13q) alone, or null for both lesions: *SF3B1* mut/*del*(13q) versus *SF3B1* wt/no del, *P* = 0.0002; *SF3B1* mut/*del*(13q) versus *SF3B1* wt/*del*(13q), *P* = 0.0002; *SF3B1* mut/*del*(13q) versus *SF3B1* mut/no *del*(13q), *P* = 0.032 (Figure 1A). These findings suggest that co-occurring *SF3B1* mutation with *del*(13q) defines a more aggressive CLL subtype with poor survival.

To determine the impact of *SF3B1* mutations in CLL with *del*(13q), we leveraged 2 murine models that allow conditional deletion of a minimal deleted region of chromosome 13q (*Mdr*) (22) or expression of *Sf3b1*-K700E (21) in B cells. A double-mutant (DM) mouse line was generated by crossing mice with an allele of *Sf3b1-K700E* and mice with a floxed (^fl/fl^) allele of *Mdr*. Then, we established a cohort of mice with B cell–specific *Mdr* deletion with (DM *Mdr-Sf3b1* MT) or without (*Mdr* MT) heterozygous *Sf3b1-K700E* by breeding the offspring with *Cd19-*Cre/Cre mice. For comparison, mice with *Cd19*-Cre/+ were included as WT controls (Figure 1B).

*Mdr* MT mice have been reported to exhibit low-penetrance CLL (22). To investigate whether the coexpression of *Sf3b1* mutation accelerates CLL in *Mdr* MT mice, we monitored the onset of CLL in 3 mouse cohorts, namely mice having B cell–specific homozygous or heterozygous deletion of *Mdr* with (DM, *n* = 25) or without (*Mdr* MT, *n* = 27) heterozygous *Sf3b1-K700E*, or lacking 2 lesions (WT, *n* = 30). We examined the appearance of typical B220^+^CD5^+^ CLL-like cells in the peripheral blood cells using flow cytometry every 3 months starting from 6 months of age up to 24 months. From 16 months onward, circulating CLL-like cells were found in 6 DM (24%) and 2 *Mdr* MT (7%) mice, with the circulating CLL burdens ranging between 20% and 60%, while no such cells were present in any WT mice (Figure 1C). Enlargement of multiple mesenteric lymph nodes was observed in 2 DM CLL mice but no *Mdr* MT CLL mice (Supplemental Figure 1A; supplemental material available online with this article; https://doi.org/10.1172/jci.insight.184280DS1).

Flow cytometry analysis and immunohistochemistry (IHC) verified the infiltration of B220^+^CD5^+^ CLL-like cells in the spleen, bone marrow, and liver (Figure 1D and Supplemental Figure 1B). CLL-like cells derived from DM or *Mdr* MT mice were further engrafted into NOD/SCID-γ (NSG) mice to determine their transplantability (Figure 1E and Supplemental Figure 1C). Mice with DM CLL cell engraftment developed a CLL-like disease within 3–4 weeks, as assessed by flow cytometry and IHC. In contrast, mice with *Mdr* MT CLL cell engraftment developed a CLL-like disease over a longer period (Figure 1E). DM CLL cells displayed more hyperproliferative markers, Ki67, and MYC compared with *Mdr* MT CLL cells (Figure 1F). Taken together, these results verify that coexpression of *Sf3b1-K700E* and *Mdr* deletion increases the penetrance of and leads to faster transplantable and aggressive CLL in vivo.

### Coexpression of Sf3b1-K700E and Mdr deletion impacts cell development and growth in normal B cells.

**Figure 2:**
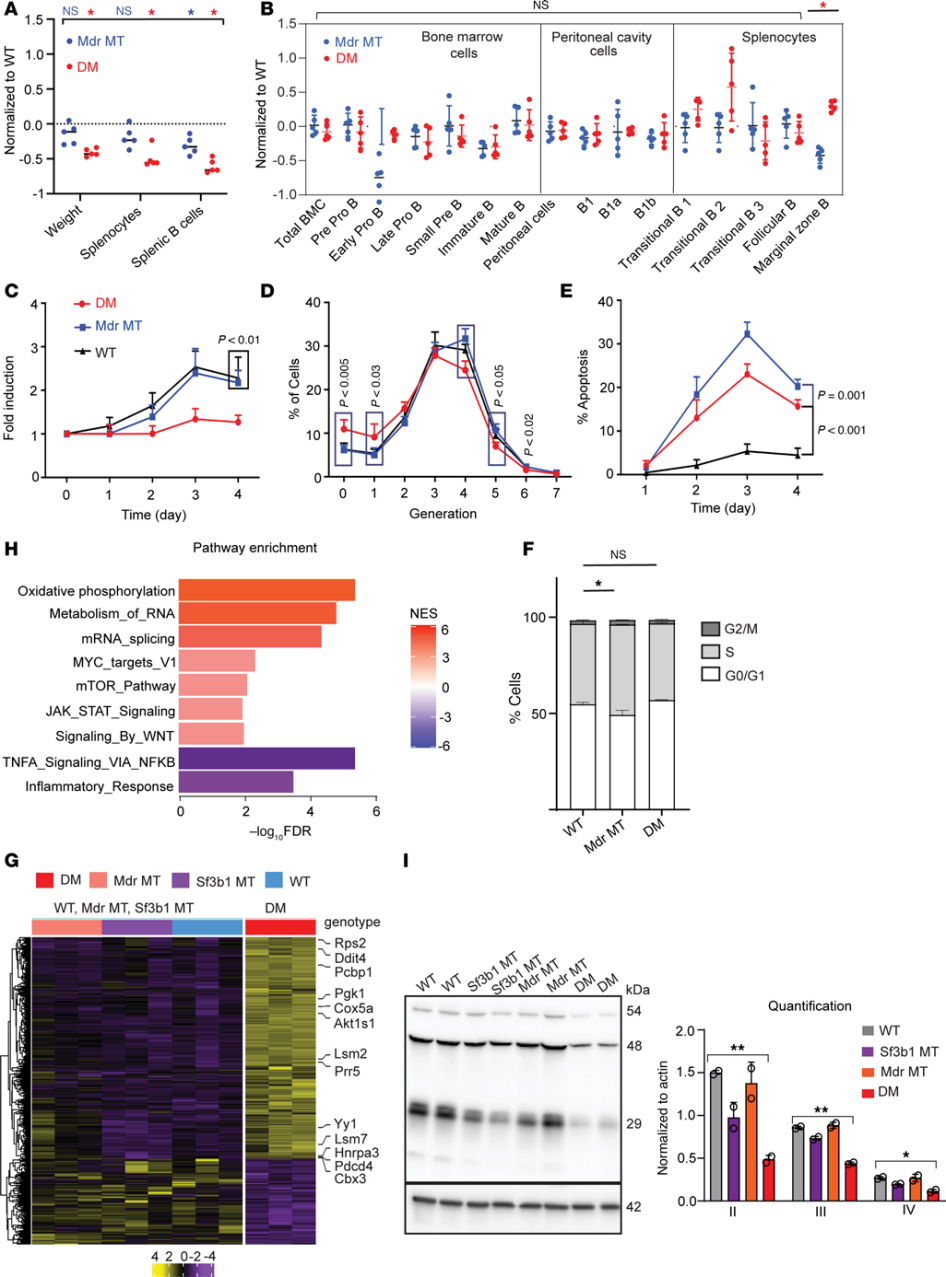
Coexpression of *Sf3b1*-K700E with *Mdr* deletion affects B cell development and growth. (**A**) Spleen weight, total number of splenocytes, and splenic B cells in WT, *Mdr* MT, and DM mice at the age of 12 weeks are shown. The fold changes in *Mdr* MT and DM mice are plotted relative to WT mice. Each dot represents 1 mouse. The center lines indicate the average. * indicates *P* < 0.01, Student *t* test. (**B**) Subsets of B cells from bone marrow and spleen in WT, *Mdr* MT, and DM mice at the age of 12 weeks are shown. The fold changes in *Mdr* MT and DM mice are plotted relative to WT mice. Each dot represents 1 mouse. Center lines indicate the average. * indicates *P* < 0.01, Student *t* test. (**C**–**F**) The proliferation curve (**C**), cell division (**D**), apoptosis (**E**), and cell cycle (**F**) of B cells were derived from WT, *Mdr* MT, and DM mice after stimulation with IL-4 and LPS in vitro. Data are presented as average ± SD and derived from 5 mice in each group except cell cycle from 3 mice in each group. Cell division and cell cycle are analyzed after stimulation for 3 days and 24 hours, respectively. *P < 0.01, 1-way ANOVA. (**G**) Heatmap shows differential gene expression between murine splenic normal B cells with DM and other genetic lesions, including *Sf3b1*-K700E or *Mdr* deletion (Log_2_FC ≥ 1, FDR < 0.05). (**H**) Gene set enrichment analysis (GSEA) of differentially expressed genes between DM cells and other cells from **G**. Significance cutoff is set as FDR < 0.1. NES, normalized enrichment score. (**I**) DM cells have reduced expression of electron transport complex II, III, IV expression in splenic normal B cells. * and ** indicate *P* < 0.01 and *P* < 0.001, respectively, Student *t* test.

To assess the impact of *Sf3b1* mutation with *Mdr* deletion on B cell biology, we evaluated cell growth, development, and proliferation in young (12-week-old) mice without CLL (Figure 2). Consistent with our observations from *Sf3b1*-K700E mice, the coexpression of *Sf3b1*-K700E and *Mdr* deletion significantly reduced spleen weight and the total number of splenocytes and splenic B cells compared with WT mice (Figure 2A and Supplemental Figure 2A). Notably, *Mdr* deletion alone also resulted in a subtle but consistent reduction in the number of splenic B cells without major changes in the weight of the spleen and the total number of splenocytes (Figure 2A). Among different subtypes of B cells in the spleen, DM increased the percentage of marginal zone B cells (*P* < 0.01, Figure 2B and Supplemental Figure 2B). In contrast, *Mdr* deletion alone decreased this subpopulation (Figure 2B), suggesting a potent role of *Sf3b1*-K700E in driving the development of marginal zone B cells, corroborating our previous results from *Sf3b1*-mutant mice (21). In both DM and *Mdr*-deleted mice, no differences were observed in other subpopulations of splenic B cells or early B cell development in bone marrow mononuclear cells and peritoneal mononuclear cells (Figure 2B and Supplemental Figure 2C).

We further evaluated B cell growth and apoptosis in response to LPS and IL-4 stimulation ex vivo. *Mdr* deletion alone did not impact cell growth but resulted in a significantly higher fraction of cells undergoing apoptosis than WT or DM cells (Figure 2, C and D). DM significantly inhibited cell growth by reducing cell division and increasing apoptosis (Figure 2, D and E), similar to our observations in *Sf3b1-K700E* mice (21). Consistent with a previous report (22), deletion of *Mdr* led to increased cycling cells (Figure 2F). In contrast, coexpression of *Sf3b1-K700E* and *Mdr* deletion did not impact the cell cycle (Figure 2F). Taken together, expression of *Sf3b1-*K700E mutation and *Mdr* deletion led to an intrinsic defect in B cells, affecting cell proliferation and apoptosis, suggesting *Sf3b1*-K700E mutation is vital in altering B cell function.

### RNA-sequencing analysis reveals enrichment of oxidative phosphorylation, MYC target genes, and mTOR pathway activation in DM normal B cells.

To elucidate the mechanism of how *Sf3b1* mutation synergistically works with *Mdr* deletion to impact B cell function, we performed RNA sequencing on RNA isolated from normal splenic B cells of mice with or without *Sf3b1* and/or *Mdr* lesions. Differential gene expression analysis of splenic B cells from DM mice (*n* = 3) compared with other genotypes (WT, *Mdr* MT, *Sf3b1* MT, *n* = 3 for each genotype) identified 835 dysregulated genes, of which 658 were significantly upregulated (Figure 2G and Supplemental Table 1). These genes were highly enriched for critical upregulated cellular pathways, including oxidative phosphorylation (OXPHOS), mRNA splicing, MYC targets, and mTOR pathway, and had downregulation of TNF-α signaling and inflammatory response (Figure 2H). Consistent with an enriched OXPHOS process, we verified that electron transport chain protein II, III, and IV expression in DM cells was significantly downregulated compared with WT cells (Figure 2I). These results indicate that expression of *Sf3b1-*K700E mutation together with *Mdr* deletion generates distinct changes in the molecular and cellular circuitry of B cells compared with the presence of a single lesion.

### Integrated transcriptomic and proteomic analysis reveals activation of mTOR complex 1 and MYC pathways in DM CLL cells.

**Figure 3:**
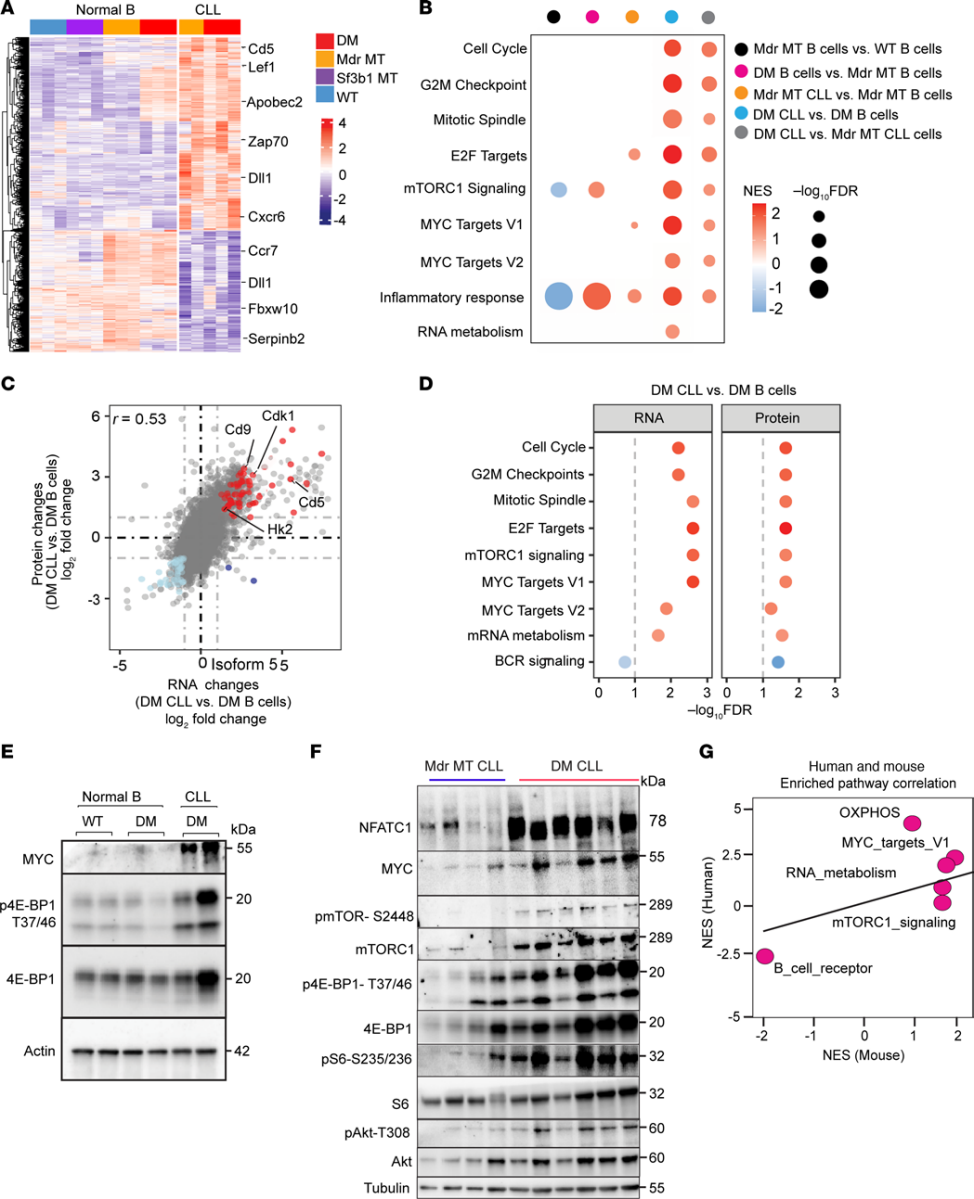
Integrative transcriptome and proteomics analyses identify enrichment of the mTOR pathway and MYC targets in DM CLL cells. (**A**) Heatmap shows significantly differentially expressed genes between CLL cells with DM or *Mdr* deletion and normal B cells with different genetic lesions, including *Sf3b1*-K700E, *Mdr* deletion, and DM. (**B**) GSEA from differentially expressed genes from different comparisons, including *Mdr* MT B cells versus WT B cells, DM B cells versus *Mdr* MT B cells, *Mdr* MT CLL cells versus *Mdr* MT B cells, DM CLL cells versus DM B cells, DM CLL cells versus *Mdr* MT CLL cells. Significance cutoff is set as FDR < 0.1. (**C**) Correlative plot of differentially expressed genes and proteins between DM CLL cells and DM normal B cells derived from RNA-Seq and proteomics data. DM CLL versus DM normal B cell fold changes are log_2_-transformed with positive and negative values indicating upregulation and downregulation, respectively. Color-coded genes are significantly differentially expressed genes at both mRNA and protein levels. (**D**) GSEA based on differential RNA and protein analysis for genes enriched for upregulated and downregulated pathways at both mRNA and protein levels. The dashed line indicates significance according to the FDR < 0.1. (**E**) Western blot of mTORC1 pathway components and MYC expression in DM CLL cells, DM and WT normal B cells. (**F**) Validation of isoform 5 of nuclear factor of activated T cells C1 (NFATC1) using *Mdr* MT CLL cells and DM CLL cells by immunoblotting. Western blot of mTORC1 pathway components, downstream targets, AKT pathway, and MYC expression in *Mdr* MT CLL cells and DM CLL cells. (**G**) Correlation plot of pathways enriched at the protein levels between human and mouse CLL with DM versus normal B cells.

To determine the transcriptome-wide changes associated with leukemogenesis in DM cells, we conducted differential gene expression analysis by comparing DM CLL cells with DM normal B cells (*n* = 3, each group). CLL cells displayed 1,059 dysregulated genes with 457 upregulated, including the well-known CLL marker gene *Cd5* (Supplemental Figure 3A and Supplemental Table 2). To further investigate the contribution of *Sf3b1* mutation and *Mdr* deletion to the oncogenesis, we also compared the gene expression of DM CLL and *Mdr* MT CLL against normal splenic B cells from different genetic groups (Figure 3, A and B). CLL cells with either *Mdr* deletion or DM both upregulated known CLL-associated genes, such as *Cd5*, *Lef1*, and *Zap70* (Figure 3A). To pinpoint the cellular processes driving the aggressiveness of CLL in DM mice, we performed GSEA using significantly differentially expressed genes derived from the following comparisons: *Mdr* MT B cells versus WT B cells, DM B cells versus *Mdr* MT B cells, *Mdr* MT CLL versus *Mdr* MT B cells, DM CLL versus DM B cells, and DM CLL versus *Mdr* MT CLL cells. GSEA suggested that *Mdr* deletion in normal B cells downregulated mTOR complex 1 (mTORC1) and inflammatory response pathways. Still, DM could override these pathways, indicating a potential synergistic role of *Sf3b1* mutation and *Mdr* lesion (Figure 3B). Consistent with previous reports (22), *Mdr* MT CLL cells displayed an upregulation of MYC and E2F targets compared with *Mdr* MT normal B cells. DM CLL cells displayed an upregulation of multiple CLL-associated pathways, including cell cycle associated (cell cycle, E2F targets, mitotic spindle, G2M checkpoint), mTORC1 signaling, and MYC target genes (Figure 3B). When comparing DM CLL cells and *Mdr* MT CLL cells, almost all the cellular pathways were highly enriched except the RNA metabolism pathway (Figure 3B and Supplemental Figure 3B), highlighting that *Sf3b1*-K700E and *Mdr* deletion synergistically contribute to the progression of CLL via the regulation of mTORC1 signaling, MYC activation, and cell cycle.

To directly query the processes involved in the leukemogenesis at the protein level, we performed an integrative transcriptomic and proteomic analysis using splenic B cells derived from DM mice with and without CLL. Differential expression analysis revealed a strong concordance between protein and mRNA levels, with a correlation coefficient of *r* = 0.5265 (Figure 3C). CLL-related genes, including *Cd5*, *Zap70*, and *Cdk9*, were significantly upregulated at the mRNA and protein levels. Similar to our observation in *Sf3b1/Atm* CLL cells (21), DM CLL cells also exhibited downregulation of B cell receptor (BCR) signaling (Figure 3D). Additionally, genes involved in MYC, cell cycle checkpoints, and mTORC1 pathways were consistently upregulated and enriched at both mRNA and protein levels (Figure 3D and Supplemental Figure 3C). Notably, we validated MYC upregulation and mTORC1 pathway activation via immunoblotting in the DM CLL cells (Figure 3E). Furthermore, we verified that DM CLL cells had a stronger impact on MYC expression and mTORC1 pathway activation compared with *Mdr* MT CLL cells, as demonstrated by increased levels of MYC protein and phosphorylated (p-) mTORC1 and its direct and indirect targets, including p-4E-BP1 T37/46, p-S6-S235/236, and p-Akt-T308 (Figure 3F), corroborating with gene expression–based pathway enrichment (Figure 3B). Importantly, mTORC1 signaling and MYC target genes were also highly enriched in human CLL cells harboring these 2 genetic lesions (23) (Figure 3G), reinforcing the notion that these pathways are central to driving the aggressiveness of CLL.

### Nuclear factor of activated T cells 1 alternative isoform leads to mTOR and MYC pathway activation.

**Figure 4:**
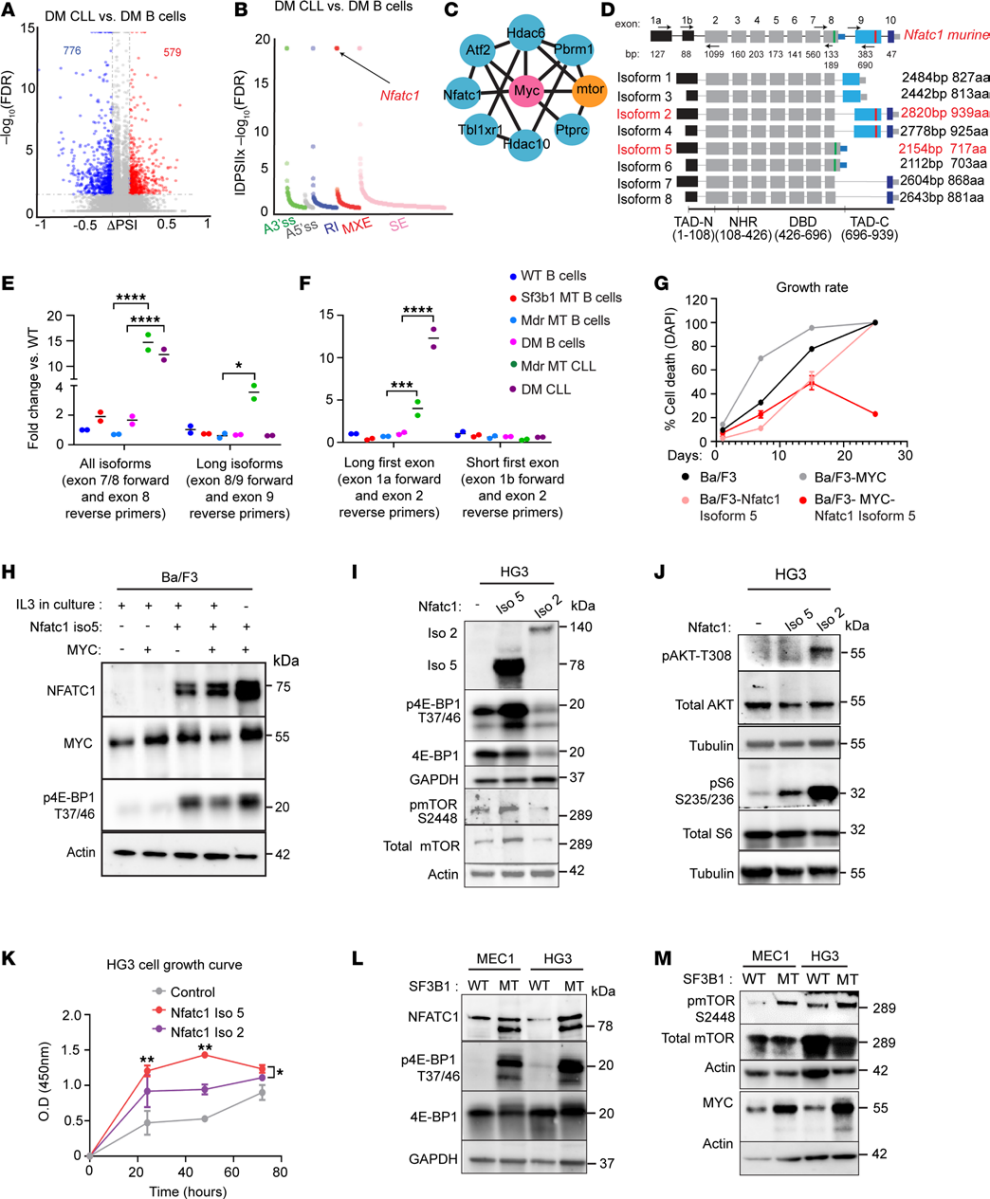
Splice variant of *Nfatc1* activates the mTOR pathway and MYC expression. (**A**) Volcano plot shows percentage of spliced-in (ΔPSI) versus log_10_ (FDR *P* value) of all splicing changes identified by rMATS between CLL and normal B cells with DM. Events with the absolute percentage of spliced-in (|ΔPSI|) > 10% and FDR < 0.05 were considered significant and color-coded. (**B**) RNA splice variants derived from **A** were plotted out as 5 different splicing types, and statistical significance was measured by the |ΔPSI| multiplied by the negative log (FDR). *Nfatc1* is one of the most consistent changed splice variants and is indicated with an arrow. (**C**) Direct interactors with mTOR and MYC were identified from overlapped genes from panel **A** based on the STRING database. (**D**) Isoforms of *Nfatc1* genes. Primers for the quantitative PCR (qPCR) were indicated with arrows. TAD-N, N-terminal transactivation domain (aa 1–108); NHR, NFAT homology region (aa 108–426); DBD, DNA-binding domain (aa 426–696); TAD-C, C-terminal transactivation domain (aa 696–939). (**E** and **F**) *Nfatc1* short isoform 5 is highly expressed in DM CLL cells measured by 2 different reverse transcription PCR assays. *P < 0.05, ***P < 0.001, ****P < 0.001; 2-way ANOVA, Šídák corrected. Data presented as SD. (**G**) *Nfatc1* isoform 5 overexpression promotes IL-3 independence in Ba/F3 cells with MYC overexpression. Dead cells were measured over 27 days with a flow cytometry–based assay upon a staggered IL-3 withdrawal. (**H**) Overexpression of *Nfatc1* isoform 5 leads to mTOR pathway activation and upregulation of MYC in Ba/F3 cells. (**I** and **J**) Overexpression of *Nfatc1* isoform 5 results in the activation of the mTOR pathway measured by phosphorylation of mTORC1, 4E-BP1, while overexpression of isoform 2 leads to the activation of AKT pathway and phosphorylation of S6 in human CLL HG3 cell line. (**K**) Overexpression of *Nfatc1* isoform 5 promotes more cell growth than isoform 2 in HG3 cells. *P < 0.05, **P < 0.01, 1-way ANOVA. Cell proliferation is measured by CCK-8 assay based on colorimetric absorbance at 450 nm over 3 days of culture. (**L** and **M**) *SF3B1* mutation promotes the expression of *NFATC1* isoform 5 and mTOR pathway upregulation in CLL cell lines HG3 and MEC1. Isogenic CLL cell lines with *SF3B1*-K700E are evaluated for NFATC1 expression, MYC and mTOR pathway activation by immunoblot.

Mutated *SF3B1* drives alternative RNA splicing and mediates the development of multiple types of tumors (11). To define the role of *Sf3b1-K700E* in the activation of mTORC1 and MYC pathways, we first identified splice variants associated with DM CLL cells as well as *Sf3b1*-K700E B cells through RNA splicing analysis using the rMATS pipeline (24) (Figure 4, A and B, and Supplemental Figure 3D). In total, 1,029 and 376 spliced genes were identified to be associated with DM CLL and *Sf3b1* mutation, respectively; 117 spliced genes overlapped between the 2 groups (Supplemental Table 3), with 7 genes having direct interaction with MYC and mTORC1 protein based on the STRING database (*Nfatc1*, *Atf2*, *Hdac6*, *Pbrm1*, *Ptprc*, *Tbl1xr1*, *Hdac10*) (Figure 4C). We selected *Nfatc1* to explore further its role in the activation of the mTOR pathway and contribution to CLL progression, as alternative splicing of *Nfatc1* displayed the highest consistent splicing changes based on *P* value and absolute percentage spliced-in value among all of these splice variants (Figure 4B). Of note, splice variants associated with DM CLL cells and *Sf3b1* mutation all resulted in BCR signaling enrichment containing the *Nfatc1* gene (Supplemental Figure 4C).

*NFATC1* is a transcription factor that plays important roles in many cellular processes, including oncogenesis (25–29). This gene produces 8 isoforms — 4 long and 4 short — by using 2 different promoters, 2 poly-A sites, and alternative splicing of exons 8 and 9 (25) (Figure 4D). In murine DM CLL cells, RNA-Seq data revealed an alternative splicing event involving exon 8 and 9, resulting in reduced expression of long isoforms (including exon 9) and increased expression of short isoforms (lacking exon 9) (Supplemental Figure 3E). Using isoform-specific qPCR assays, we identified isoform 5 as the predominant isoform expressed in DM CLL cells (Figure 4, E and F). Expression of total *Nfatc1* was assessed using primers spanning exons 7 and 8, while long isoform expression was measured using primers targeting the exon 8–9 junction. While total *Nfatc1* expression levels appeared comparable between *Mdr* MT and DM CLL cells, the short isoform lacking exon 9 was more prevalent in DM CLL cells (Figure 4E). In addition, exon 1a was preferentially utilized in DM CLL cells (Figure 4F). Protein-level validation confirmed the expression of the 78 kDa short isoform 5 in DM CLL cells (Figure 3F). Collectively, these findings demonstrate that *Nfatc1* isoform 5 is preferentially expressed in DM CLL cells, whereas isoform 2 predominates in *Mdr* MT CLL cells.

Different isoforms of *NFATC1* have been previously explored for their oncogenic roles (28, 30, 31). Particularly, isoforms lacking the C-terminal TAD have been reported to promote proliferation and oncogenic activity due to the absence of the TAD’s pro-apoptotic function and the activation of MYC pathway via epigenetically transcriptional regulation (30–33). To explore the oncogenic potential of isoform 5, we overexpressed this isoform in Ba/F3 and Ba/F3 MYC cells. Of note, Ba/F3 is a murine pro–B cell line commonly used for oncogene screening because of its dependency on IL-3, while Ba/F3 MYC cells are utilized for screening weak oncogenes (34). Isoform 5 expression led to MYC upregulation, activation of the mTOR pathway, and enhanced cell growth (Figure 4, G and H). Remarkably, this isoform conferred IL-3 independence in Ba/F3 MYC cells, consistent with its previously reported role in B cells (Figure 4G).

To further evaluate isoform-specific effects, we overexpressed isoform 5 and full-length *Nfatc1* (isoform 2, as a control) in the human CLL HG3 cell line (Figure 4I). As expected, isoform 5 was associated with the mTOR pathway, evidenced by increased phosphorylation of mTORC1 and 4E-BP1. In contrast, isoform 2 inhibited the activation of these proteins but induced activation of the AKT pathway and S6 protein (Figure 4J), suggesting that the 2 isoforms engage distinct signaling cascades. Consistent with these findings, both isoforms promoted cell growth, with isoform 5 exerting a more pronounced effect (Figure 4K). Taken together, our results suggested that alternative RNA splicing of *Nfatc1*, driven by *Sf3b1* mutation, contributes to the aggressiveness of CLL through isoform-specific activation of mTOR signaling.

### Murine DM CLL cells recapitulate human CLL cells with SF3B1 mutation and del(13q).

Given our murine DM CLL cells displayed the activation of the mTOR pathway and MYC upregulation, we further investigated these findings in human CLL. We examined RNA-Seq and proteomics data from publicly available datasets (24, 35, 36). Differential gene expression between CLL cells with and without *SF3B1* mutation/*del*(13q) indicated the upregulation of the MYC target pathway and downregulation of the inflammatory pathway (Supplemental Figure 4A), consistent with the role of these 2 lesions in the murine model. Proteomics data further revealed the significant enrichment of MYC targets, metabolism of RNA, and OXPHOS, along with a positive tendency for the mTOR pathway (Supplemental Figure 4B).

Next, we examined RNA splicing events associated with *SF3B1* mutation/*del*(13q). Comparison of CLL cells with *SF3B1* mutation/*del*(13q) to CLL cells either with *del*(13q) or *SF3B1* mutations revealed enrichment for mTORC1 pathway and BCR signaling associated with *SF3B1* mutation but not *del*(13q) (Supplemental Figure 4C). Alternative RNA splicing of *NFATC1* was one of the splice variants detected in human CLLs with *SF3B1* mutation when compared with CLL without *SF3B1* mutation, in the presence or absence of *del*(13q), based on RNA-sequencing data (Supplemental Table 4). Of note, with our newly established *SF3B1*-mutant isogenic CLL cell lines (37), we confirmed that expression of *SF3B1* mutation generates upregulated *NFATC1* isoform 5 expression and increased MYC and the activation of the mTOR pathway by the detection of p-4E-BP1 and mTORC1 (Figure 4, L and M). Taken together, our results verified that murine DM CLL cells recapitulate human CLL cells with *SF3B1* mutation and *del*(13q).

### Targeting the mTORC1 pathway and RNA splicing is beneficial to DM CLL in vitro and in vivo.

**Figure 5:**
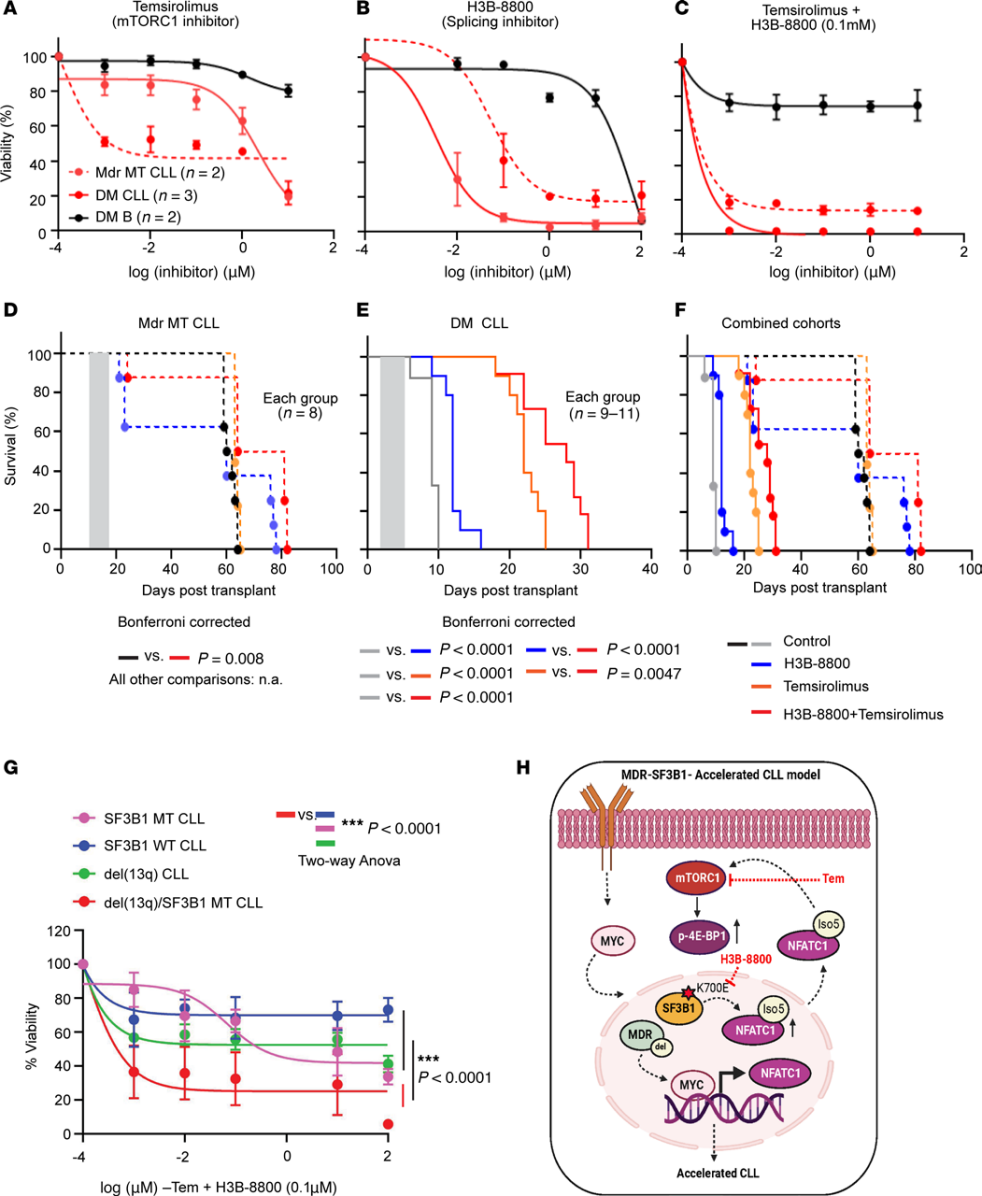
Targeting RNA splicing and the mTORC1 pathway has therapeutic effects in DM CLL cells. (**A**–**C**) Cell viability of *Mdr* MT CLL, DM CLL, and DM normal B cells treated with either H3B-8800 or temsirolimus or in combination over 24 hours. (**D**–**F**) Survival curve of *Mdr* MT CLL mice, with DM CLL mice treated with either single-drug H3B-8800 (4 mg/kg, 5 days, oral gavage) or temsirolimus (15 mg/kg, 5 days, intraperitoneal injection) or in combination. Gray shaded area indicates treatment time. CLL mice were established by engrafting *Mdr* MT CLL or DM CLL cells into NSG mice. Treatment started with detectable 3%–5% circulating CLL based on flow cytometry. (**G**) Cell viability of human CLL cells with or without *SF3B1* mutation, in the presence or absence of *del*(13q) after 24 hours of treatment with increasing concentrations of temsirolimus (0.1 nM to 10 μM) combined with 0.1 mM H3B-8800. Black dotted line indicates the dosage used for statistical calculation. Two-way ANOVA with Tukey’s test was used to compare *del*(13q) with *SF3B1* mutation with other groups. (**H**) Schematic summary of the mechanism of DM CLL development.

Given that *SF3B1* mutation and *del*(13q) activate the mTORC1 pathway through RNA splicing, we hypothesized that DM CLL cells are sensitive to either RNA splicing inhibitor or mTORC1 inhibitor treatment. As a proof of concept, we selected the targeted pathway inhibitors H3B-8800 and temsirolimus (Tem) to target RNA splicing and the mTORC1 pathway, respectively. We exposed murine DM B (*n* = 3) DM CLL (*n* = 2), as well as *Mdr* MT CLL (*n* = 2) cells to either Tem or H3B-8800 or in combination for 24 hours and then measured the cell viability by CellTiter-Glo assay. Compared with normal B cells, *Mdr* MT CLL and DM CLL cells responded to Tem, H3B-8800, and their combination (Figure 5, A–C). *Mdr* MT CLL cells were more sensitive to Tem treatment (IC_50_: 0.0001055 μM) compared with DM CLL cells (IC_50_: 2.148 μM), possibly due to these cells relying more on cap-dependent translation, which is inhibited by Tem, leading to reduced protein synthesis even in the absence of high mTORC1 activity (Figure 5A). Conversely, DM CLL cells were more sensitive to the splicing inhibitor H3B-8800 (IC_50_: 0.00346 μM) compared with *Mdr* MT CLL cells (IC_50_: 0.05331 μM) (Figure 5B), consistent with the known role of H3B-8800 in targeting *SF3B1* mutations. Furthermore, a more pronounced combinatorial effect was found in DM CLL cells when H3B-8800 was combined with Tem, consistent with a potential synergistic interaction based on the Chou-Talalay combination index (38), where a combination index (CI) < 1 indicates synergy and CI = 1 indicates an additive effect (*Mdr* MT CLL versus DM CLL: 1.96 × 10^–4^ versus 3.56 × 10^–6^) (Figure 5C). Taken together, our results implicated that RNA alternative splicing and mTORC1 activation might interact with each other to drive DM CLL progression.

We then tested the effects of these drugs in vivo. For the in vivo drug efficacy test, *Mdr* MT and DM CLL cells were engrafted into NSG mice, and different drug treatments were started when the CLL cell percentage in the lymphocyte population reached 3%–5%. In *Mdr* MT CLL mice, treatment with Tem (15 mg/kg, i.p., 5 days) or H3B-8800 (4 mg/kg, oral gavage, 5 days) alone did not affect survival, but the combined treatment did improve survival (*P* = 0.0088, log-rank test) (Figure 5D). In DM CLL mice, both single and combined treatments significantly impacted overall survival (*n* = 9–11, each group, all comparisons *P* < 0.01, log-rank test) (Figure 5E). Notably, the synergistic effect in DM CLL mice was more pronounced, with a median overall survival of 27 days compared with 9 days in the control group (3-fold increase). In contrast, in *Mdr* MT CLL mice, the median overall survival for combined treatment was 63 days, compared with 61 days in the control group (1.05-fold increase) (Figure 5F). Consistently, we also observed a reduction in spleen size and CLL cell percentage in the lymphocyte population within peripheral blood, splenocytes, and bone marrow cells in the combined drug treatment group after 5 days of exposure to the treatment in DM CLL mice (Supplemental Figure 5, A–C). As expected, H3B-8800 and Tem single-drug treatment could reduce the expression level of 4E-BP1 and p-4E-BP1 T37/46 in the splenic cells in each group (Supplemental Figure 5D). In contrast, we detected only a slight reduction of spleen weight in *Mdr* MT CLL mice (Supplemental Figure 5E). These in vivo data strongly support that targeting RNA splicing and mTOR pathways significantly improves overall survival in DM CLL mice.

To determine whether H3B-8800 and Tem combination treatment could be translated to human CLL, we exposed CLL patient samples with both *del*(13q) and *SF3B1* mutations (*n* = 3) or without these 2 lesions (*n* = 3) or with *del*(13q) alone (*n* = 3), or with *SF3B1* mutations alone (*n* = 3) to a series of concentrations of Tem (0.1 nM to 10 μM) combined with H3B-8800 (0.1 mM) in vitro for 24 hours. We then examined the cell viability with an ATPase-based CellTiter-Glo assay. DM CLL cells were more sensitive to this combination treatment than all the other 3 groups (*P* < 0.001, 2-way ANOVA) (Figure 5G), corroborating our observation in murine CLL cells, highlighting that RNA splicing and mTOR pathways are essential for DM CLL cells.

## Discussion

Our studies highlight the significant impact of coexpressing *Sf3b1*-K700E and *Mdr* deletion in murine B cells and their role in CLL biology, with emphasis on how these 2 genetic lesions cooperate to alter B cell development, growth, and CLL progression.

One of the key observations is the higher penetrance of CLL in murine models when both *Sf3b1*-K700E and *Mdr* deletion are coexpressed compared with each mutation alone. This finding is consistent with the clinical data from patients with CLL, where *SF3B1* mutations and *del*(13q) frequently co-occur and are associated with inferior overall survival and faster disease progression. This correlation reinforces the relevance of the murine model and its potential implications for understanding CLL biology in human patients and providing a test bed for effective therapies in CLL patients with these lesions.

The integrated transcriptomic and proteomic analysis of DM CLL cells provides valuable insights into the mTORC1 and MYC pathways involved in the aggressiveness of CLL (Figure 5H). The upregulation of MYC targets and the alteration of RNA metabolism point toward a critical role of *SF3B1* in regulating alternative RNA splicing and subsequently impacting vital cellular processes to regulate the aggressiveness of CLL. In particular, our study implicates *Nfatc1* as a candidate mediator of mTOR pathway activation in DM CLL cells. Notably, *Nfatc1* is known to be upregulated in human CLL and murine CLL models, which acts downstream of BCR signaling and promotes CLL survival (26, 30, 39, 40). Consistent with this, we observed that *SF3B1* mutation led to upregulation of NFATC1 in isogenic HG3 cell lines and murine CLL cells (Figure 4, E and L). Our findings further showed that isoform-specific expression of *NFATC1* activates distinct signaling pathways, resulting in differential effects on cell proliferation. This strongly suggests that alternative splicing represents an additional, previously underappreciated regulatory layer that fine-tunes downstream signaling and contributes to the aggressive phenotype of CLL.

*SF3B1* mutation accelerates cancer progression through alternative RNA splicing in various cancer types by activating the Akt and NF-κB pathway, MYC activation, inflammation pathway, and TGF-β signaling in several cancers. In 3 disease types — pancreatic ductal adenocarcinoma (14), UVM (12), and lymphoma (16) — the MYC pathway is activated through alternative splicing in *PPP2R5A* or *BRD9*. However, in other cancer types (breast cancer and MDS), alternative splicing in *PPP2R5A*, *MAP3K7*, and *IRAK4* has been linked to the activation of Akt and NF-κB pathway, TGF-β signaling, and inflammation pathway, respectively. Notably, the same splice variant, *PPP2R5A*, can lead to disease progression by activating different cellular pathways. These results highlight that *SF3B1* mutation–induced splice variants contribute to cancer progression in a cellular context– and disease-dependent manner, underscoring the need to assess the functional impact of *SF3B1* mutations tailored to specific diseases. As we demonstrated here, *SF3B1* mutation activates the mTOR pathway through alternative splicing of *Nfatc1*, linking *SF3B1* mutation, BCR signaling, and the mTOR pathway in CLL and providing potential therapeutic options specific to the disease context.

The therapeutic implications of this study are particularly significant. H3B-8800, an orally available small-molecule splicing modulator, combined with temsirolimus targeting the mTORC1 pathway, showed promising results in reducing the viability of DM CLL cells in vitro and in vivo. The observed additive effect from this combination treatment suggests a potential synergistic impact when these pathways are inhibited simultaneously. While H3B-8800 has demonstrated effectiveness in spliceosome-mutant cancers (41), its use was limited due to toxicity observed in phase I clinical trial (42), leading to its current unavailability on the market. Our findings suggest that combination therapy might be viable, as lower doses of H3B-8800 could effectively treat the disease. Further extensive testing of different combinations will be crucial to explore this possibility fully.

Overall, this study enhances our understanding of the role of *SF3B1* mutations and *del*(13q) in CLL biology and provides valuable insights into the complex interplay of genetic lesions in B cell development and CLL progression. The findings provide potential avenues for targeted therapies and personalized treatment approaches for CLL patients with specific genetic profiles. However, further research and validation in larger patient cohorts are needed to translate these findings into clinical applications and improve patient outcomes. Additionally, investigating targeted therapies’ potential side effects and long-term efficacy is crucial to ensure their safety and effectiveness in clinical settings.

## Methods

### Sex as a biological variable.

We studied both male and female animals and found similar results in both. Human samples from males and females also showed similar findings.

### Human samples.

Peripheral blood cells were isolated by density gradient centrifugation using Ficoll-Paque Medium (GE Healthcare, now Cytiva). Normal B cells were isolated by immuno-magnetic negative selection with a pan–B cell isolation kit (Miltenyi Biotec). All samples were cryopreserved with fetal bovine serum (FBS) 10% DMSO and stored in vapor-phase liquid nitrogen until analysis.

### Cell lines and reagents.

Leukemia cell lines HG3 (provided by Richard Rosenquist, Karolinska Institutet, Stockholm, Sweden), MEC1 (ACC497, DSMZ), and Ba/F3 with or without MYC expression (provided by David Weinstock, Dana-Farber Cancer Institute) were cultured in RPMI 1640 medium (Invitrogen) supplemented with 10% FBS and 1% penicillin/streptomycin. All cell lines were incubated at 37°C with 5% CO_2_, authenticated by short tandem repeat analysis, and determined as mycoplasma-free before being used for experiments.

Antibodies used in this study include anti-phosphorylated mTOR (2855, Cell Signaling Technology), anti-mTOR (2983, Cell Signaling Technology), anti-MYC (D84C12, Cell Signaling Technology), anti-Annexin V (640906, BioLegend), anti-NFATC1 (MA3-024, Thermo Fisher Scientific), anti-GAPDH (sc-365062, Santa Cruz Biotechnology), and anti-Actin (Santa Cruz Biotechnology). Secondary antibodies include Goat anti-rabbit IgG secondary antibody, HRP (65-6120, Invitrogen), and Goat anti-mouse IgG secondary antibody, HRP (65-6520, Invitrogen). HRP activity was revealed using Clarity or Clarity Max ECL Western Blotting Substrates (1705061 or 1705062, Bio-Rad). Temsirolimus was purchased from LC Laboratories, and H3 Biomedicine Inc. provided H3B-8800.

### Animals.

*Sf3b1*-K700E–floxed mice (C57BL/6J × 129 hybrids) were generated as previously reported (21). *Mdr*-floxed mice (22) (C57BL/6J × 129 hybrids) were ordered from The Jackson Laboratory. To obtain heterozygous expression of *Sf3b1* mutations and heterozygous *Mdr* deletion in B cells, we crossed *Sf3b1*-K700E–floxed mice (21) with *Mdr*-floxed mice (22) to generate *Sf3b1*^fl/+^ *Mdr*^fl/fl^ mice, which were then crossed with *Cd19*-Cre/Cre to obtain DM mice (*Cd19*-Cre/+ *Sf3b1*^fl/+^ *Mdr*^fl/+^). To obtain heterozygous expression of *Sf3b1* mutation and homozygous *Mdr* deletion in B cells, we crossed *Sf3b1^fl/+^ Mdr^fl/fl^* mice with *Mdr^fl/fl^ CD19-Cre* (*Cd19*-Cre/Cre) mice to obtain DM mice (*Cd19-Cre^fl/+^* *Sf3b1^fl/+^ Mdr^fl/fl^*) and Mdr-mutant (*Cd19-Cre^+/–^ Sf3b1^+/+^ Mdr^fl/fl^*) mice.

### Murine model and disease monitoring.

Approximately 100 μL of blood was collected via submandibular bleeding into EDTA-coated tubes. A total of 1 mL of ACK buffer was used for erythrocyte lysis and then washed with PBS with 1% BSA and 2 mM EDTA (FACS buffer). Cells were then stained with a cocktail of antibodies: CD5 (PE/Cy5 anti-mouse CD5 [clone 53-7.3], BioLegend), B220 (Pacific Blue anti-mouse/human CD45R/B220 [RA3-6B2], BioLegend), CD3 (APC/Cy7 anti-mouse CD3 [17A2], BioLegend), CD11b (PE/Cy7 anti-mouse/human CD11b [M1/70], and Ig Kappa (Alexa Fluor 700, BioLegend) for 15 minutes at 4°C. Cells were further washed with FACS buffer and analyzed by flow cytometry. All flow cytometry assays were performed on a LSRFortessa (BD Biosciences).

### B cell subpopulation analysis.

The proportion of the cell subpopulations from the spleen, bone marrow, and peritoneal cavity was analyzed by flow cytometry based on the expression of surface markers, as we previously reported (21). Cells from the spleen or the bone marrow were extracted by mechanical dissociation, and erythrocyte lysis was carried out by osmotic lysis. Briefly, the cell pellet was resuspended in 9 mL of water to allow erythrocyte lysis. Then, 1 mL of 10× PBS was immediately added, followed by 20 mL of FACS buffer to stop the lysis. Cells from the peritoneal cavity were collected by peritoneal lavage. All cells were washed in FACS buffer and incubated with the corresponding antibody cocktail for 15 minutes at 4°C. Samples were subjected to a flow cytometer analysis after washing them once with the FACS buffer.

Cell suspensions prepared from the spleen were stained with the following antibodies: anti-B220-Pacific Blue [RA3-6B2], anti-CD93-BV605 [AA4.1], anti-CD23-APC, anti-CD21-PE [7E9], anti-IgD-APC-Cy7 [11-26c.2a], and anti-IgM-PE-Cy7 [RMM-1] for marginal zone B cell, follicular B cell, and transitional B cell quantification; marrow cell suspensions were stained with anti-B220-Pacific Blue [RA3-6B2], anti-CD43-APC [S11], anti-CD24-FITC [M1/69], anti-IgM-PE-Cy7 [RMM-1], anti-IgD-APC-Cy7 [11-26c.2a], and anti-Ly51-PE [6C3] for pro–B cell, pre–B cell, immature B cell, transitional B cell, and mature B cell quantification; and peritoneal cavity cells were stained with anti-CD5-PE-Cy5 [clone 53-7.3], anti-B220-Pacific Blue [RA3-6B2], and anti-CD11b-PE-Cy7 [M1/70] antibodies for B1a cell quantification. All the antibodies are from BioLegend.

### B cell functional evaluation.

Mice were euthanized in a CO_2_ chamber, and spleens were harvested and mechanically dissociated to form a single-cell suspension. Erythrocyte lysis was carried out by osmotic lysis, and B cells were immuno-magnetically selected from the single-cell suspension using the MACS B cell isolation kit for mice (Miltenyi Biotec). After sorting B cell purity was confirmed using the flow cytometry staining for at least 85% pure but typically more than 90%. B cells were cultured in RPMI 1640 supplemented with 10% FBS, 0.1% IL-4, 0.1% 2-Mercaptoethanol (Thermo Fisher Scientific), and 50 μg/mL LPS (Sigma-Aldrich) at a cell density of 0.5–0.8 × 10^6^/mL at the start point for up to 96 hours. Every 24 hours, cell numbers were recorded, cell division was analyzed using Cell Trace Violet Cell Proliferation Kit (Thermo Fisher Scientific), and apoptosis was measured using PE Annexin V Apoptosis Detection Kit (Thermo Fisher Scientific). The cell cycle was measured using the Click-&-Go Plus EdU 647 Flow Cytometry Assay Kit (Thermo Fisher Scientific).

### IHC staining.

Freshly isolated spleens and bone marrow tissues were fixed in neutral formalin overnight and replaced with 70% ethanol the next day until the tissues were processed. Spleens were paraffin-embedded, and 10 μm sections were made for IHC staining. Ki67, CD5, B220, and cMYC levels were estimated by respective antibodies as reported (21) and HRP-conjugated secondary antibody to reveal the diaminobenzidine (DAB) staining. IHC stains were performed on DISCOVERY Ultra (Ventana Medical Systems, Roche Diagnostics) IHC Auto Stainer. Briefly, the FFPE tissue blocks were sectioned at a thickness of 5 μm and put on positively charged glass slides. The slides were loaded on the machine, and deparaffinization, rehydration, endogenous peroxidase activity inhibition, and antigen retrieval were first performed. Then, each primary antibody was incubated, followed by DISCOVERY anti-Rabbit HQ or DISCOVERY anti-Mouse HQ and DISCOVERY anti-HQ-HRP incubation. The stains were visualized with DISCOVERY ChromoMap DAB Kit, counterstained with hematoxylin (Ventana), and coverslipped. IHC image analysis was done using the Visiopharm tool. A total of 4 areas were randomly chosen and quantified using in-house developed apps in the tool following the manufacturer guidelines.

### CLL transplant.

The transplantation studies were performed on 8–12 weeks of immunodeficient recipient NSG mice using viably cryopreserved splenocytes from DM CLL or *Mdr* MT CLL animals. A total of 3 million splenocytes from CLL animals were intravenously injected via tail to NSG mice for passageability evaluation; blood sampling and flow cytometer analysis were performed for disease monitoring every 2 weeks.

### Drug treatment in vitro.

Mouse CLL cells were collected from the spleen of NSG mice engrafted with CLL cells. Normal B cells are enriched by immunomagnetic beads using the method mentioned above. Cells were seeded in 96-well plates in RPMI 1640 supplemented with 10% FBS, 0.1% IL-4, and 0.1% 2-Mercaptoethanol and cultured in 96-well tissue culture plates (50,000 cells/100 μL). Tem (LC Laboratories) and H3B-8800 (H3 Biomedicine Inc.) were diluted serially in a medium and were added to corresponding wells with the final concentrations ranging from 0 to 10 μM. After incubation for 24 hours at 37°C with 5% CO_2_, cell viability was measured by CellTiter-Glo–based luminescence assay and normalized by cells with DMSO treatment.

### Drug treatment in vivo.

For the in vivo study, Tem stock solution was dissolved in ethanol at 50 mg/mL and stored at –20°C. On the day of injections, the stock was diluted in 5% Tween 80, 5% polyethylene glycol-400 (Sigma), and PBS to the appropriate final concentration. H3B-8800 was dissolved in DMSO at 10 mM stored at –20°C and further diluted in 10% Tween 80, 10% ethanol, and 80% saline to the appropriate final concentration. A total of 1 million DM CLL or *Mdr* MT CLL cells/recipient were resuspended in 100 μL of PBS and injected intravenously into NSG mice of 8–12 weeks of age. After the CLL burden in the peripheral blood reached 5%, confirmed by flow cytometer, NSG mice were randomly assigned into 4 groups to receive the following treatment: control group, Tem treatment group (15 mg/kg/d, intraperitoneal injection), H3B-8800 treatment (4 mg/kg/d, oral gavage), and combination treatment group with both Tem and H3B-8800. The drug treatment was performed for 5 days, and then animals were observed for survival; criteria for euthanasia included hunched posture, difficulties breathing or moving, visible hepatosplenomegaly, and weight loss equal to 20% body weight — the first day when the drug treatment started was indicated as day 1. CLL burden was evaluated by flow cytometer analysis of blood samples on days 1 and 5. On the last day of drug treatment, blood samples were collected 3 hours after H3B-8800 treatment, RNA was extracted from the blood sample, and qPCR was performed to investigate the efficacy of H3B-8800 on RNA splicing inhibition using *Slc15a19* and *Dph2* as the target genes, as previously reported (41).

### Human CLL drug treatment.

*Del*(13q) CLL primary patient samples with or without *SF3B1* mutation identified by FISH and next-generation sequencing were obtained from the CLL Research Consortium. CLL samples were suspended in RPMI 1640 medium supplemented with 10% FBS and 0.1% IL-4 and seeded in 96-well tissue culture plates (50,000 cells/100 μL). Tem and H3B-8800 were diluted serially in a medium and were added at final concentrations ranging from 0 to 10 μM. After incubation for 24 hours, cell viability was measured by CellTiter-Glo–based luminescence assay. Cell viability was obtained by normalizing cells with DMSO drug treatment.

### RNA sequencing, data processing, differentially expressed mRNA analysis, and differentially expressed mRNA splicing analysis.

Normal splenic B cells or CLL B cells were first enriched by a pan-B cell selection kit (Miltenyi Biotec), and total RNA was isolated from these cells using a Nucleospin RNA plus kit (MACHEREY-NAGEL). Libraries for RNA sequencing were constructed using the Stranded Total RNA Prep with Ribo-Zero Plus Kit (Illumina) and sequenced on the NovaSeq S4 platform (Illumina) using paired-end 150 bp mode. The FASTQ sequence files exported by the sequencer were checked using FastQC (http://www.bioinformatics.bbsrc.ac.uk/projects/fastqc). Adaptors and low-quality bases were removed from the sequencing reads using Trimmomatic (43). The remaining reads were aligned to the mouse reference genome (mm10) using STAR (20) with default parameters. Adaptor trimming and mapping quality reports were generated using MultiQC (44). The DESeq2 (45) R package performed differential expression mRNA analyses. mRNAs with absolute log_2_FC more than 1 and FDR less than 0.05 were identified as significantly dysregulated genes.

RNA splicing analysis was performed using our previously established pipeline (24, 46). In brief, we integrated StringTie (47), LeafCutter (48), and rMATs (49) to maximally improve the power of detection of splicing dysregulation. We assembled de novo transcripts using StringTie with default parameters. LeafCutter was used to detect additional novel exon boundaries. Together with the isoform annotation file downloaded from GENCODE (release 26), we merged all isoform information to generate a comprehensive isoform annotation file using a custom R script as a reference file for rMATs. Percentage spliced-in value was calculated using rMATs. For differential splicing analysis, we adopted the differential splicing analysis statistical model from rMATs with an absolute IncLevelDifference value of more than 0.1 and FDR less than 0.05 as significant cutoff. Detailed differentially expressed genes and splice variants are listed in Supplemental Tables 1–5. GSEA was conducted using a preranked gene list derived from differential expression analysis. Databases from the Molecular Signatures Database, specifically the HALLMARK, KEGG, and Reactome collections, were employed. Gene sets achieving an FDR < 0.1 were considered significantly enriched pathways.

For the semiquantitative measurement of transcription, 2 μg of total RNA was reverse-transcribed using a high-capacity cDNA synthesis kit (Invitrogen) with random hexamers following the manufacturer’s instructions. A total of 1 μL of cDNA was analyzed using Quant Studio qPCR machine (Applied Biosystems, Thermo Fisher Scientific), and transcript levels were quantified using the 2^(–ΔΔCt)^ method. The primers used in this study are listed in the Supplemental Table 5.

### Tandem mass tag proteomics sample preparation, liquid chromatography–mass spectrometry, and data analysis.

CLL and normal splenic B cells were lysed with triethylammonium bicarbonate buffer supplemented with protease inhibitors and PMSF. A total of 300 μg lysates were precipitated and digested to obtain peptides. Tandem mass tag (TMT) 10-plex labeling was performed, and peptides were fractionated via basic pH reversed-phase HPLC. An 1100 pump (Agilent) equipped with a degasser and a photodiode array detector (Thermo Fisher Scientific) was used. Peptides were subjected to a linear gradient from 3% to 25% acetonitrile in 0.125% formic acid using an Agilent 300 Extend-C18 column and were fractionated into 96 fractions. Mass spectrometry was performed using an Orbitrap Fusion mass spectrometer (Thermo Fisher Scientific) coupled to a Proxeon EASY-nLC 1000 liquid chromatography pump (Thermo Fisher Scientific). Peptides were detected (MS1) and quantified (MS3) in the Orbitrap. MS2 spectra were searched using the SEQUEST algorithm against a UniProt composite database derived from the mouse proteome containing its reversed complement and known contaminants. Peptide spectral matches were filtered to a 1% FDR using the target-decoy strategy combined with linear discriminant analysis. The detected proteins were filtered to a = 200 and an isolation specificity of 0.5. Statistical proteome analysis was performed based on the normalized intensities of the TMT-reporter ions.

The peptide and protein abundance from TMT proteomics data were log_2_-transformed. Mean protein intensity was calculated among technical replicates. Proteins detected in all samples were retained for downstream analysis. For differential protein expression analysis between CLL and normal B cells, we used a previously established method with minor modifications (50). The size factor, according to the total loading for each sample, was first calculated to normalize the total amount of detected peptides. Log_2_-transformed protein intensities were normalized by quantile normalization for all samples. Differentially expressed proteins were identified using the LIMMA linear model methodology (51).

### Western blot.

Cells were lysed in RIPA buffer (Thermo Fisher Scientific) supplemented with a protease-phosphatase cocktail (Pierce Protease and phosphatase inhibitor minitablets EDTA-free, Thermo Fisher Scientific) for 30 minutes at 4°C before sonication, and protein quantification was measured using BCA assay (Pierce, Thermo Fisher Scientific). A total of 20 μg protein was separated on SDS-PAGE (4%–15% Criterion Precast Gel, Bio-Rad Laboratories) and transferred to nitrocellulose membrane (Trans-Blot Turbo nitrocellulose membranes, Bio-Rad Laboratories). Membrane strips were blocked in 5% BSA in TBS–0.1% Tween 20 and incubated overnight at 4°C with respective primary antibodies. Then, membranes were washed 3 times with TBS–0.1% Tween 20 and incubated for 1 hour with goat anti-mouse/rabbit secondary antibodies (Thermo Fisher Scientific 62-6520 and 65-6120). Subsequently, the membranes were developed for ECL detection (Clarity Western ECL substrate, Bio-Rad Laboratories) following the manufacturer’s instructions. Images were acquired using ChemiDoc MP (Bio-Rad Laboratories). Protein bands were quantified using Bio-Rad Laboratories imaging software (Image Lab 6.1).

### Lentiviral transduction and overexpression of mouse Nfatc1 isoforms in mouse and human cell lines.

HEK293T-lentiX cells, utilized for lentivirus production, were cultured in DMEM supplemented with 10% FBS. Cells were plated at a density of 3 × 10^5^ cells per well in a 6-well plate and allowed to adhere overnight. Transfections were done using Polyethyleneimine (PEI Max 40K, catalog 24765, Polysciences). The components were mixed in the following ratio: 4 parts sgRNA/overexpression construct, 2 parts pVSVG, and 3 parts psPAX2. The mixture was prepared in Opti-MEM (Life Technologies, Thermo Fisher Scientific). At 48 hours posttransfection, the viral supernatants were collected and filtered through a 0.45 μm Nalgene syringe filter SFCA (Whatman). The virus was then concentrated by ultracentrifugation using 38.5 mL tubes (344058, Beckman Coulter) at 22,000*g* for 2 hours at 4°C. Cells, at a density of 2 × 10^5^, were spin-transduced with 10–20 μL of the concentrated virus at 37°C and 2,200*g* for 90 minutes using polybrene (8–10 μg/mL, MilliporeSigma). After transduction, cells were washed with PBS and resuspended in fresh RPMI medium containing 10% FBS and 1% penicillin-streptomycin. This was done 24 hours after the transduction to promote recovery and expression. Full-length mouse *Nfatc1* and short isoform were csubloned in the pHIV-Zsgreen construct, and lentiviruses were prepared to transduce the Ba/F3/Ba/F3-MYC/HG3 cell line.

### Statistics.

Statistical analysis was performed using GraphPad Prism 9.3.1. A 2-tailed Student *t* test was used to compare the 2 groups. For more than 2 groups, *P* values were calculated with a 1-way or 2-way ANOVA test followed by a post hoc Dunnett’s, Tukey’s, or Šídák’s multiple-comparison test. A *P* value less than 0.05 was considered statistically significant. The type of statistical test used and the results, including *P* value, means, median, and standard error of the mean, are shown in the figures and figure legends.

### Study approval.

Heparinized blood samples were obtained from healthy donors and patients enrolled on clinical protocols with written informed consent, approved by the Human Subjects Protection Committee of the City of Hope (IRB#18067, IRB#06229) or Dana-Farber Cancer Institute.

All animals were housed at the City of Hope National Medical Center (COH), Duarte, California, USA. All animal procedures were completed in accordance with the *Guidelines for the Care and Use of Laboratory Animals* (National Academies Press, 2011). All protocols were approved by the Institutional Animal Care and Use Committee at COH (IACUC 17071).

### Data availability.

Values for all data points in graphs are reported in the Supporting Data Values XLS file. All the murine RNA-sequencing data are deposited in GEO (GSE300699). Human RNA-sequencing data are from CLL map (https://cllmap.org/). Murine proteomics data are available upon request from corresponding author.

## Author contributions

BZ, PI, and LW designed research studies and analyzed the experimental data. BZ, PI, ETH, ZJC, MF, and KLH conducted experiments. MJ analyzed RNA-sequencing and proteomics data. KS and DN performed the clinical correlation. LR, EMG, and TJK provided clinical samples. RC, WCC, and JYS reviewed all the IHC slides. YH, CJW, and LW supervised the studies. BZ, PI, and LW wrote and revised the manuscript, with all authors contributing to the editing.

## Supplementary Material

Supplemental data

Unedited blot and gel images

Supplemental table 1

Supplemental table 2

Supplemental table 3

Supplemental table 4

Supporting data values

## Version 1 07/22/2025

In-Press Preview

## Version 2 09/09/2025

Electronic publication
