# Supplementary material for: SF3B1 mutation accelerates the development of CLL via activation of the mTOR pathway
Source: JCI Insight. 2025 Jul 22;10(17):e184280. doi: 10.1172/jci.insight.184280 (PMC12487679; doi:10.1172/jci.insight.184280)
Supplement: Supplemental data [file jciinsight-10-184280-s031.pdf]

Supplemental information

SF3B1 mutation accelerates the development of CLL via activation of the mTOR pathway

Bo Zhang[1,2☆], Prajish Iyer[1☆], Meiling Jin[1☆], Elisa ten Hacken[3☆], Zachary J. Cartun[3], Kevyn L. Hart[1], Mike Fernandez[1], Laura Rassenti[4], Emanuela M. Ghia[4], Thomas J. Kipps[4], Ruben Carrasco[3], Wing C. Chan[5, 6], Joo Y. Song[5, 6], Yu Hu[2], Catherine J. Wu[3], Lili Wang[1,6]

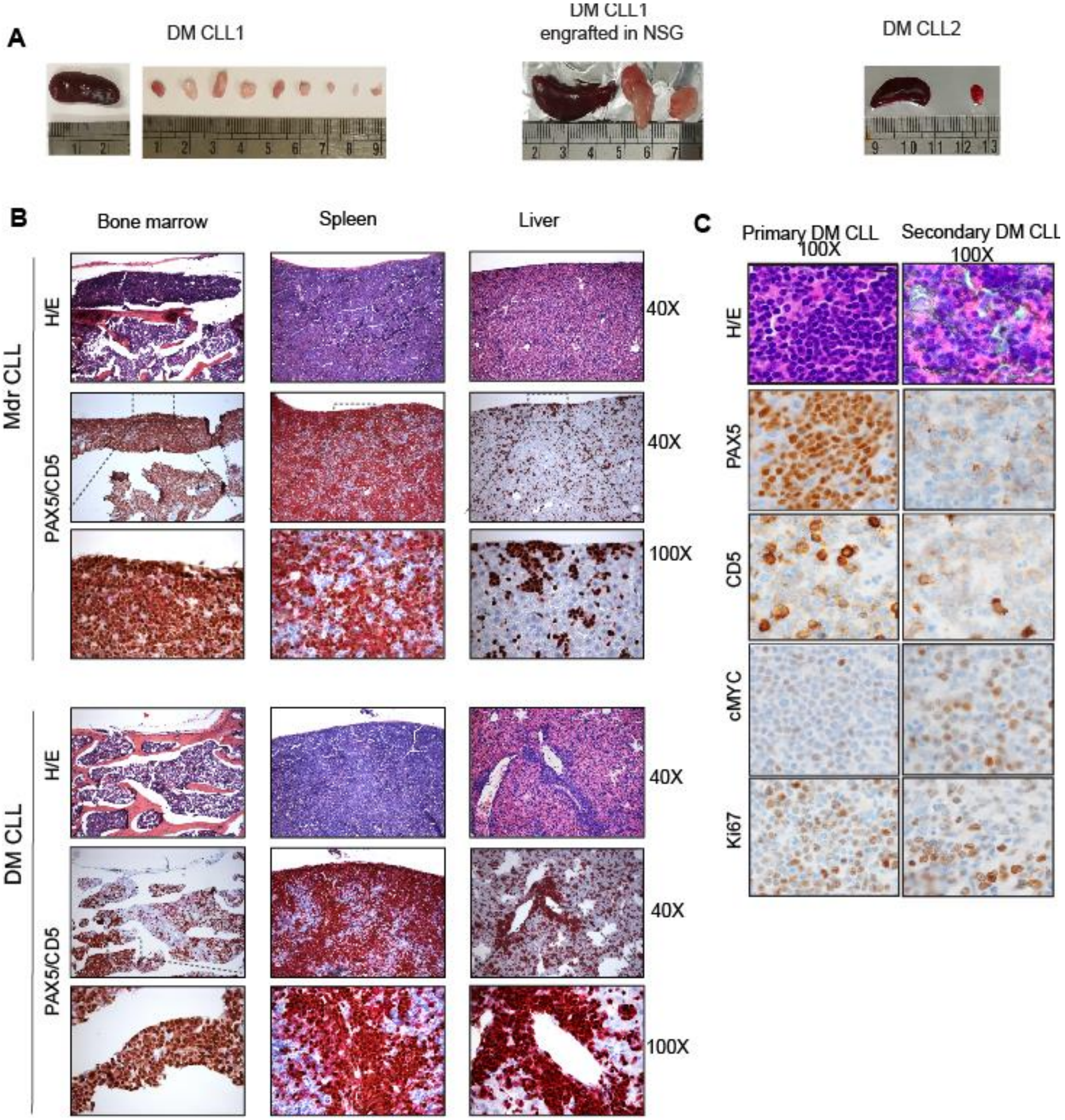

Supplemental Figure 1. Co-expression of *Sf3b1*-K700E and *del*(13q) in murine B cells leads to aggressive CLL. (A) Images of spleens and lymph nodes from two DM CLL mice. (B) Immunohistochemical staining of PAX5/CD5 and H&E on bone marrow, spleen, and liver sections derived from *Mdr* MT CLL and DM CLL mice. (C) Immunohistochemical staining of PAX5, CD5, MYC, and Ki67 along with H&E on sections of spleen derived from primary and secondary DM CLL mice.

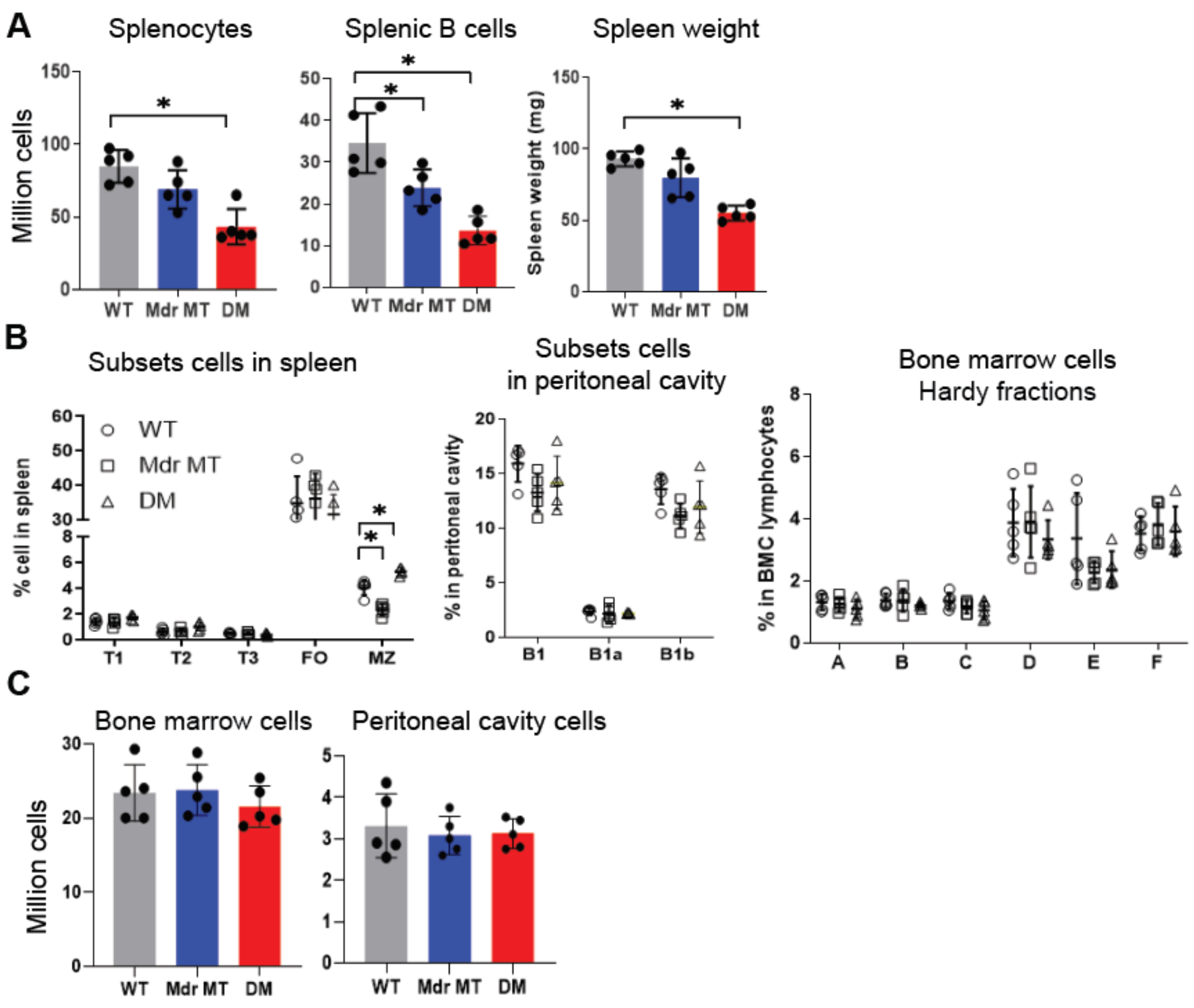

Supplemental Figure 2. Co-expression of *Sf3b1*-K700E with *Mdr* deletion impacts B cell development. (A-C) Spleen, bone marrow, and peritoneal cavity cells were collected from WT, *Mdr* MT, and DM mice at the age 12 weeks. Each dot represents one mouse. (A, C) Spleen weight, total splenocytes, splenic B cells, total bone marrow cells, and total peritoneal cavity cells were measured and plotted. * and ** indicate p< 0.01 and p<0.001, respectively. Student *t*-test. (B) Subsets of B cells from spleen, peritoneal cavity, and bone marrow in WT, *Mdr* MT, and DM mice were profiled by flow cytometry. Percentage of subsets of cells were plotted. * Indicates p< 0.01. Student *t*-test.

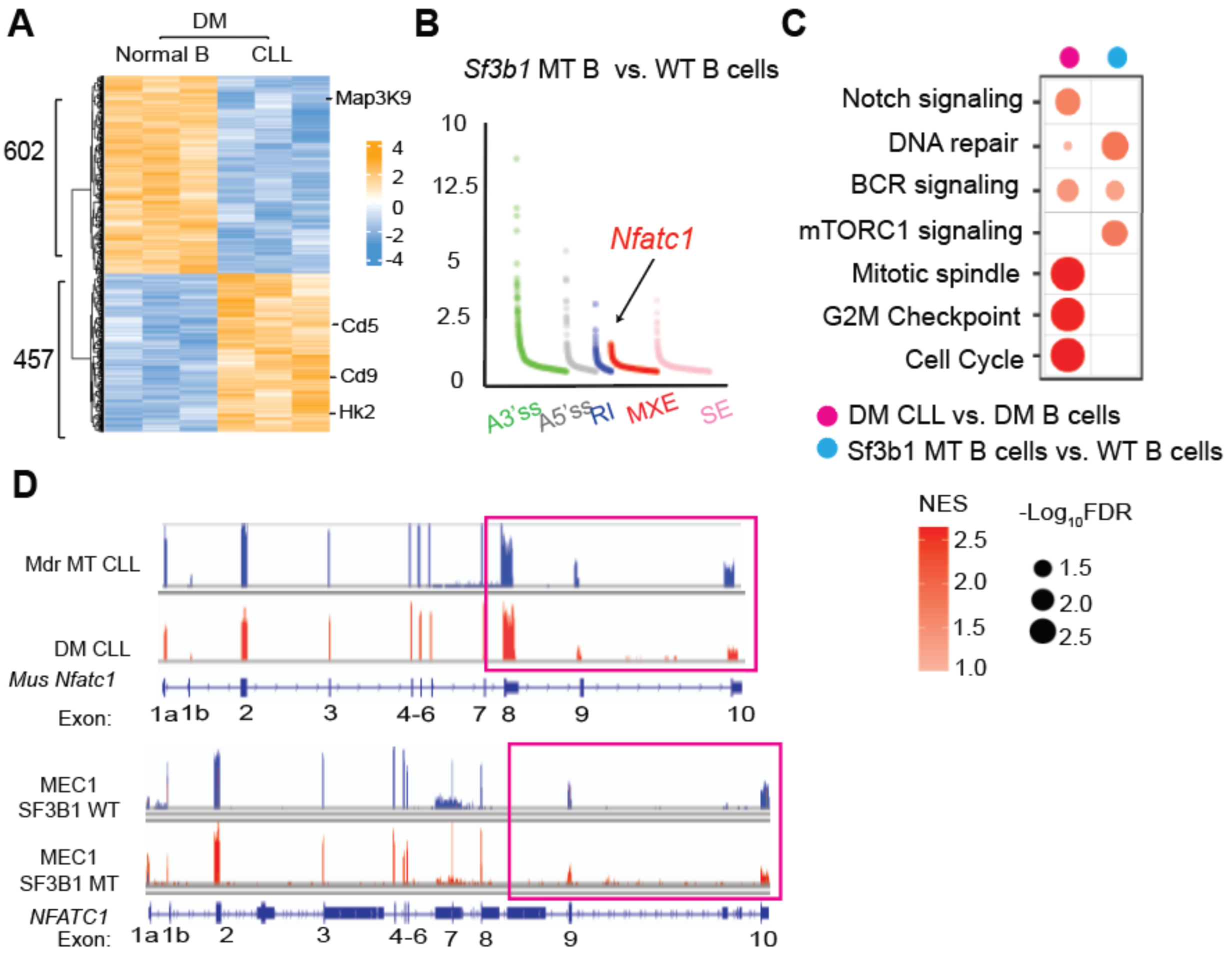

Supplemental Figure 3. Co-expression of *Sf3b1*-K700E with *Mdr* deletion impacts gene expression and RNA splicing in CLL cells. (A) Differential gene expression between DM CLL cells and DM normal B cells in heatmap. (B) RNA splice variants derived from *Sf3b1* mutant B cells vs. WT B cells. Data was plotted out as 5 different splicing types and statistical significance was measured by the absolute percentage of spliced-in (PSI) multiple by the negative log (FDR). (C) GSEA analysis of genes with significant splice variant changes associated with murine DM CLL cells or *SF3B1* mutation in normal B cells. NES indicates normalized enrichment score. Only gene sets with FDR < 0.1 were plotted. (D) *NFACT1* gene IGV track of RNA-seq data from DM CLL cells, *Mdr* MT CLL cells, MEC1 *SF3B1* WT and MT cell lines. Magenta box indicates exon 9 and 10 of *NFATC1*.

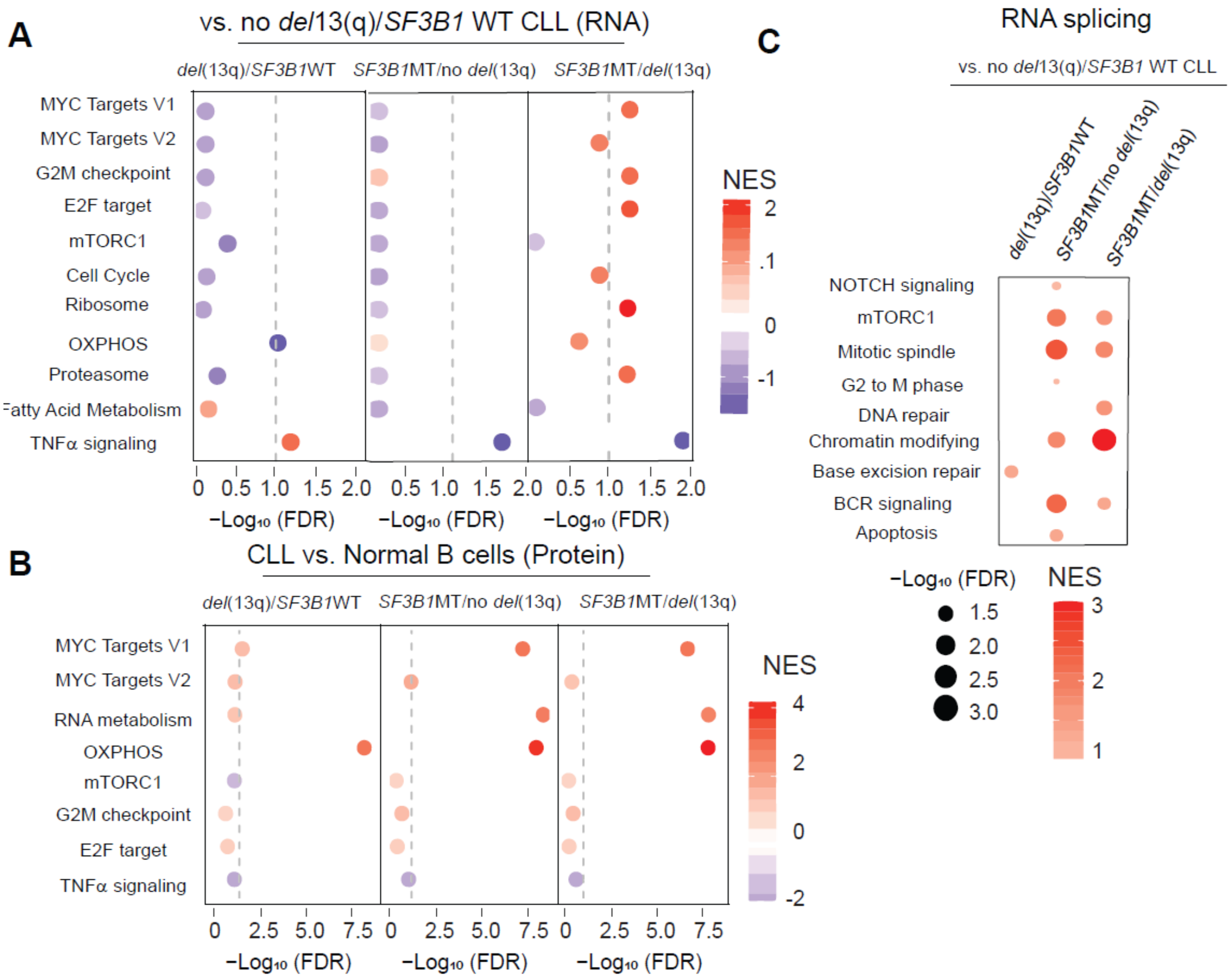

Supplemental Figure 4. Co-expression of *Sf3b1*-K700E with *Mdr* deletion impacts gene and protein expression, as well as RNA splicing. (A, C) Differential gene expression and RNA splice variants were identified between CLL with the indicated genotypes vs. CLL cells without *del*(13q) and *SF3B1* mutations. Enriched cellular pathways are shown. Data are all from the CLL map (https://cllmap.org/). (B) Differential protein expression identified upregulation of MYC targets, RNA metabolism, and OXPHOS. We reanalyzed all the data from previous publications using our pipelines (Meier-Abt, F et al. Blood 2021, Wu, Y et al. Blood Cancer Discovery 2023).

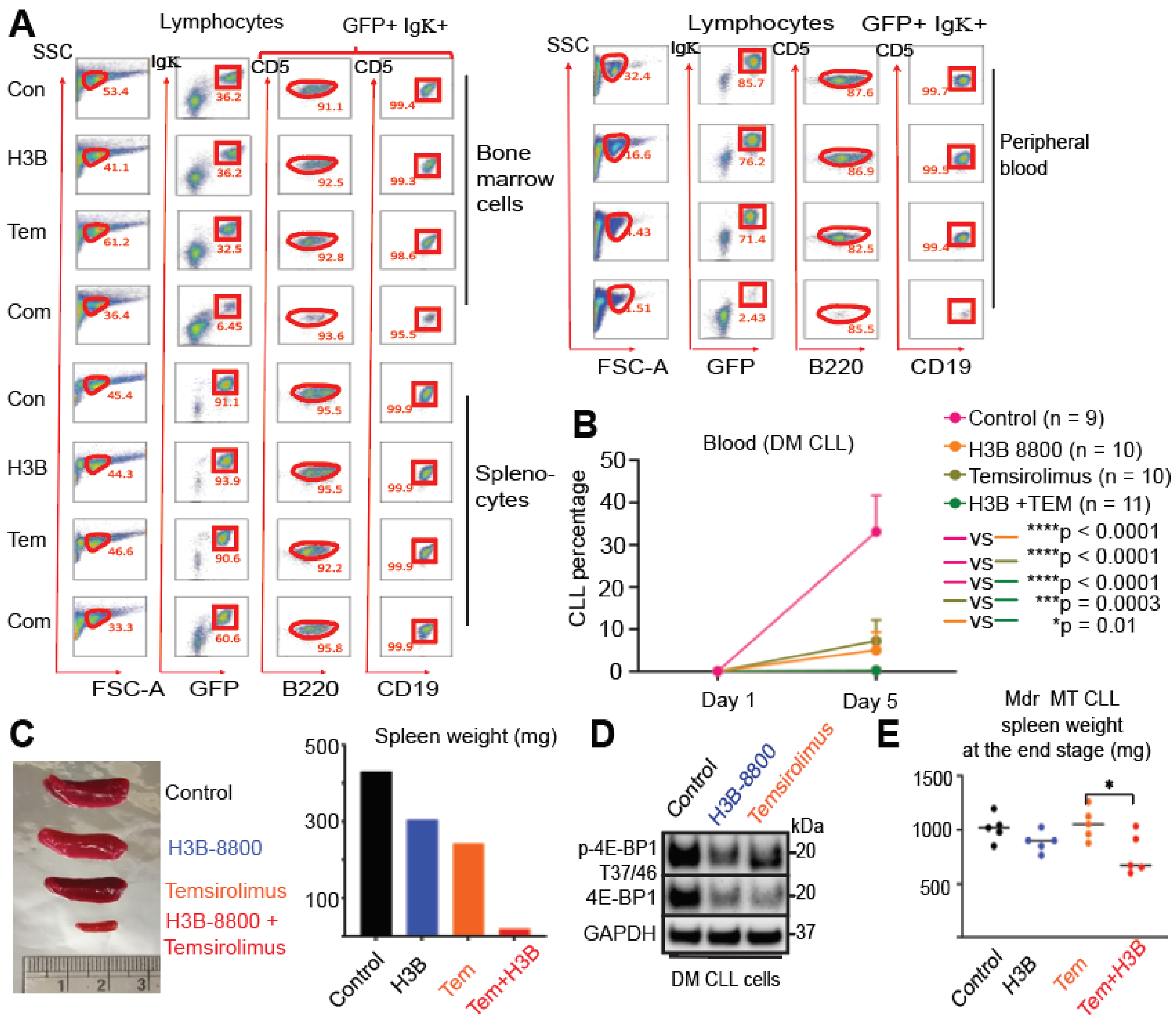

Supplemental Figure 5. Targeting RNA splicing and mTOR pathway in *Mdr* MT CLL and DM CLL mice. (A) DM CLL mice were sacrificed 5 days post drug treatment. CLL disease at the bone marrow, spleen, and peripheral blood were profiled by flow cytometry. (B) CLL disease was profiled from peripheral blood in the DM CLL mice with different drug treatment 5 days post treatment. (C) Spleen was imaged and spleen weight was measured in the different treatment group. (D) 4E-BP1 total and phosphorylated form were probed in splenocytes from the drug treatment group. (E) Spleen weight was measured and plotted at the end stage of *Mdr* MT CLL mice from different treatment groups.
