# Supplementary material for: SF3B1 mutation accelerates the development of CLL via activation of the mTOR pathway
Source: JCI Insight. 2025 Jul 22;10(17):e184280. doi: 10.1172/jci.insight.184280 (PMC12487679; doi:10.1172/jci.insight.184280)

Figure 2I- Uncropped raw image

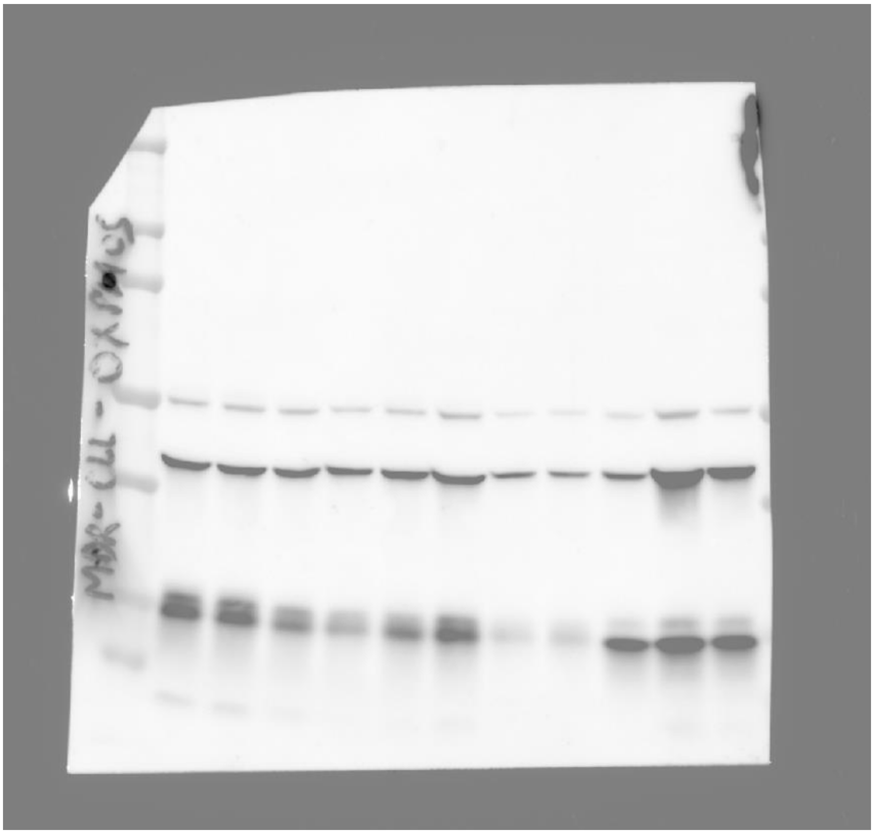

OXPHOS COMPLEX

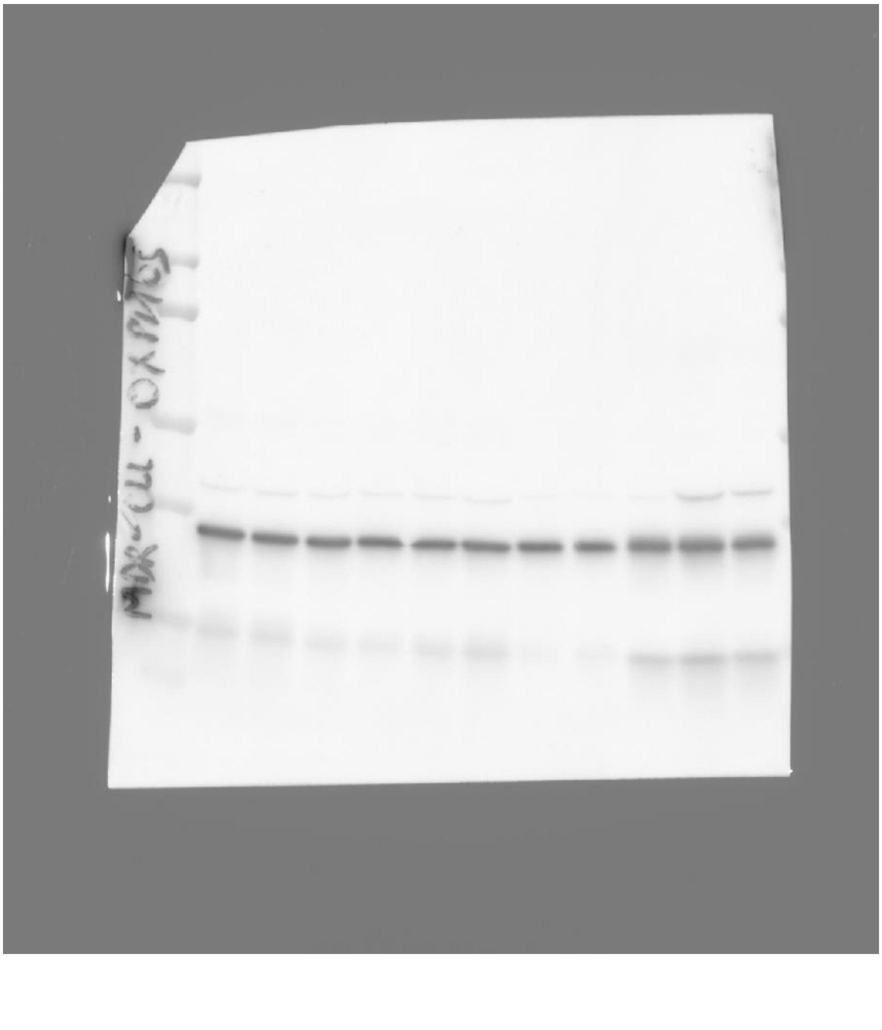

Actin

Updated- Figure 3E- Uncropped raw image

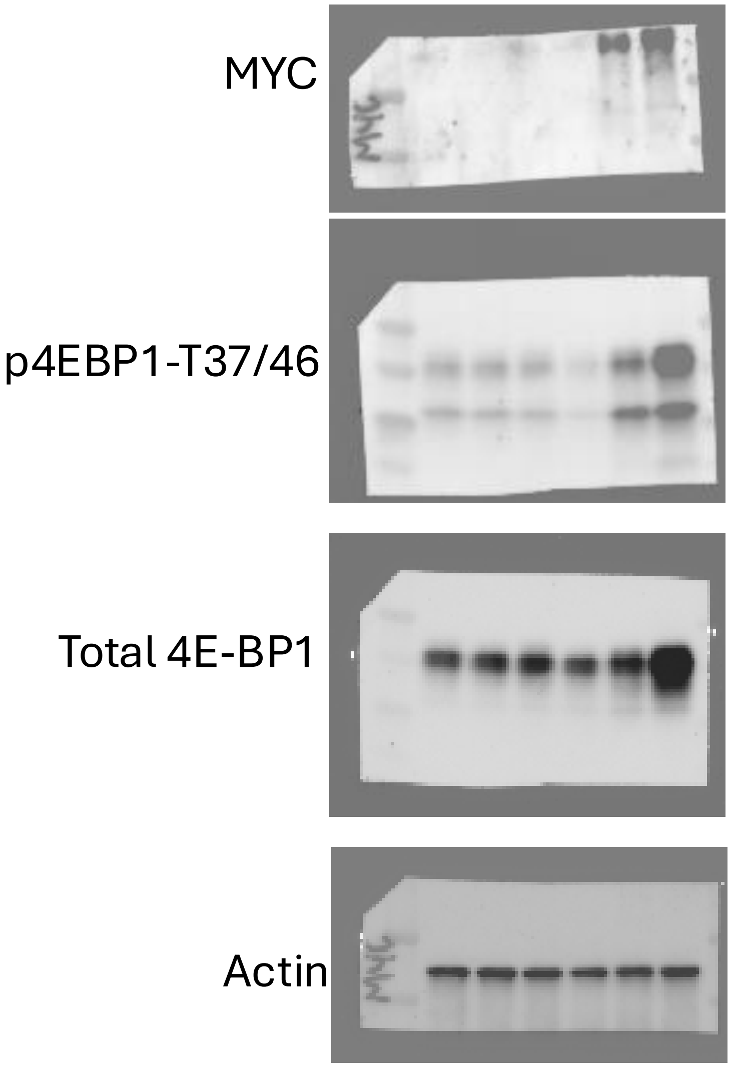

Figure 4I- Uncropped raw image

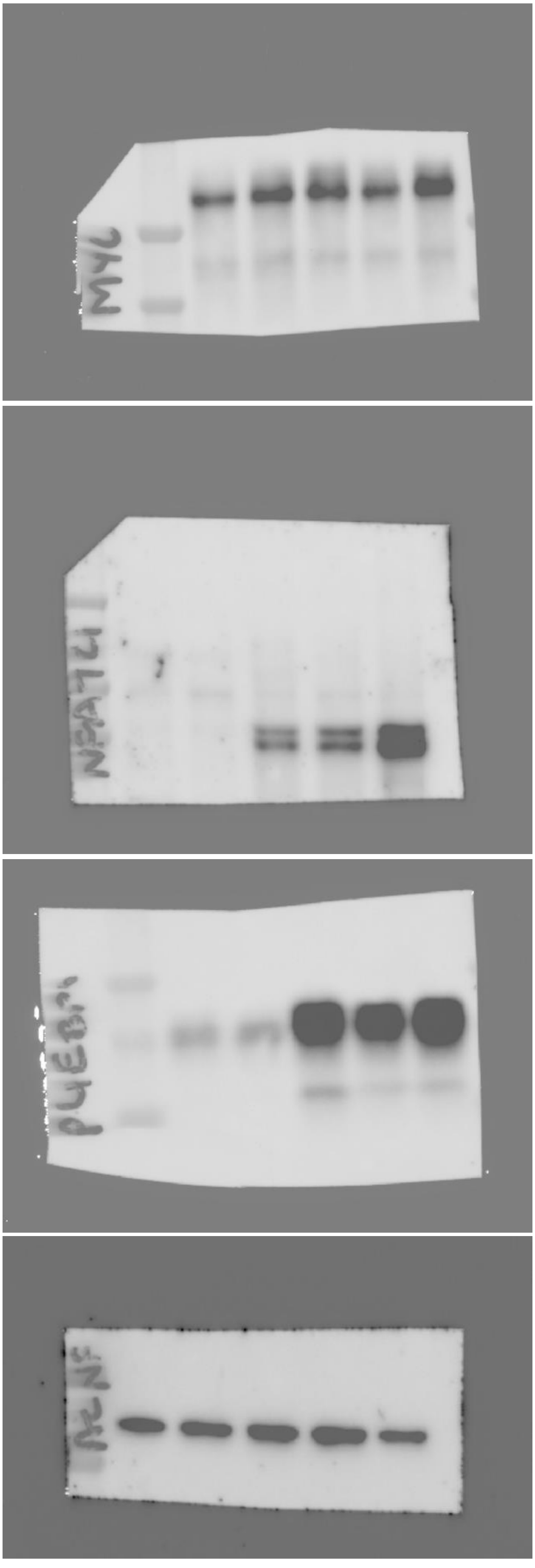

MYC

NFATC1 Isoform 5

p4E-BP1 T37/46

Actin

Figure 4J- Uncropped raw image

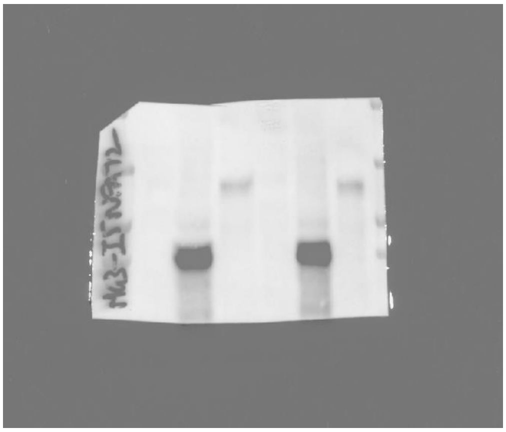

NFATC1

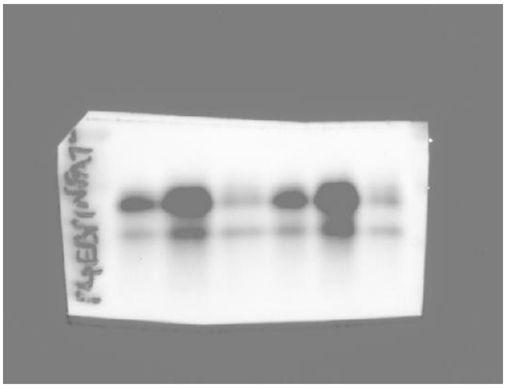

p4EBP1

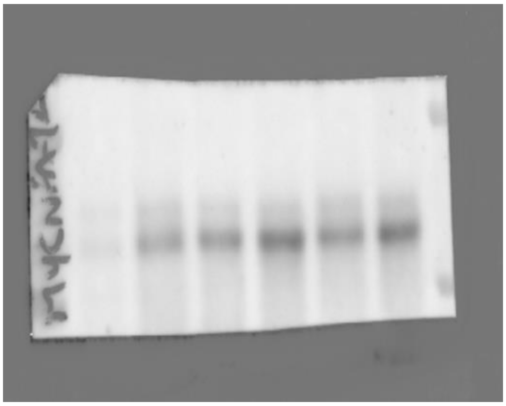

MYC

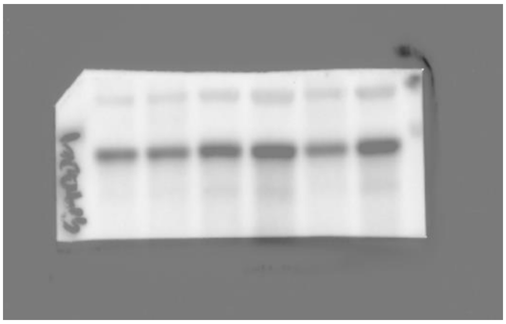

GAPDH

Fig 5F Uncropped images

p4E-BP1

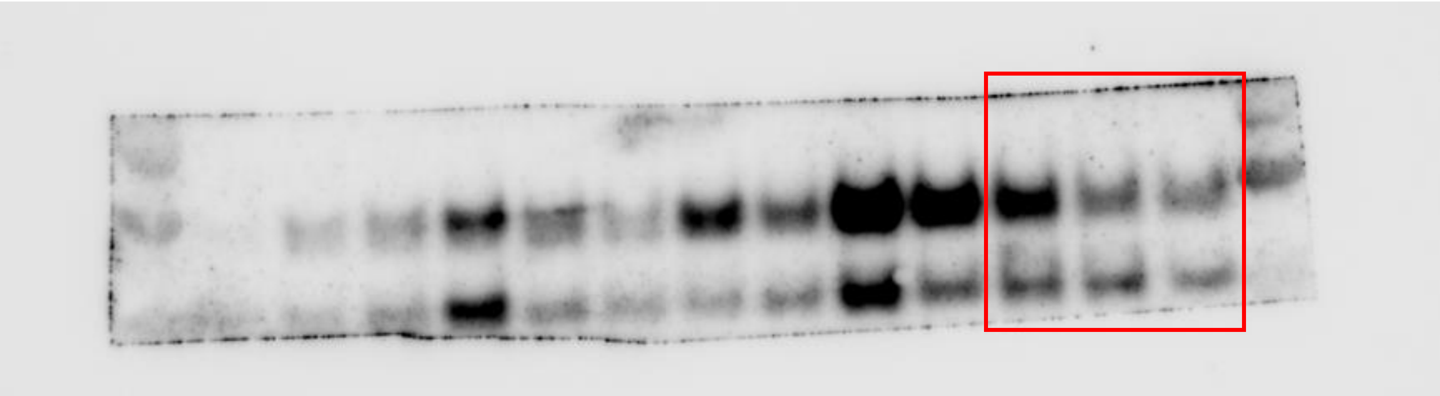

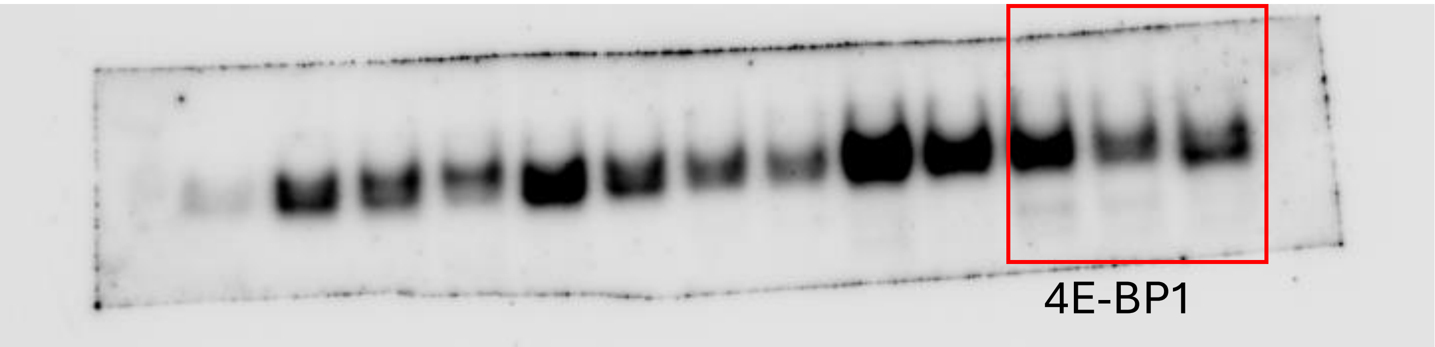

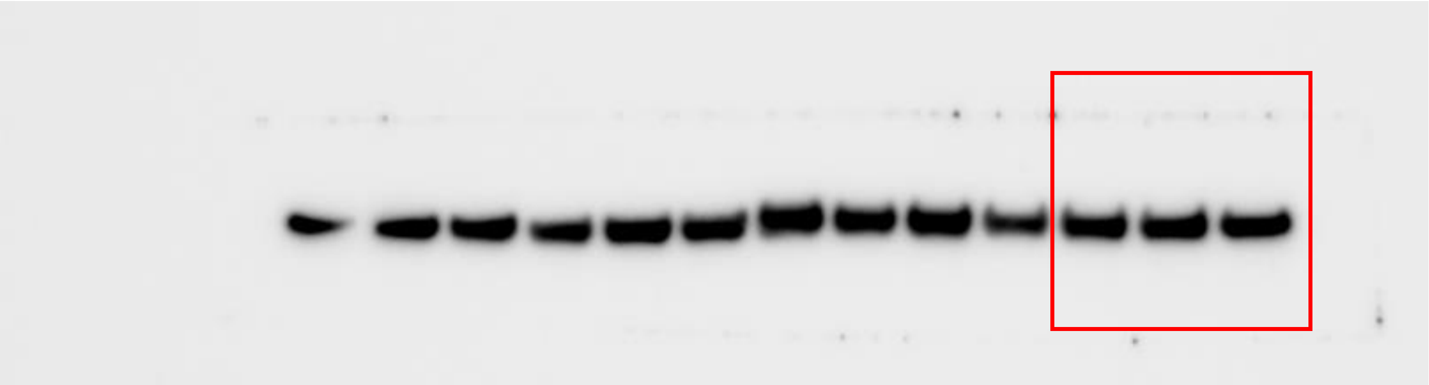

GAPDH

Figure 3F_updated

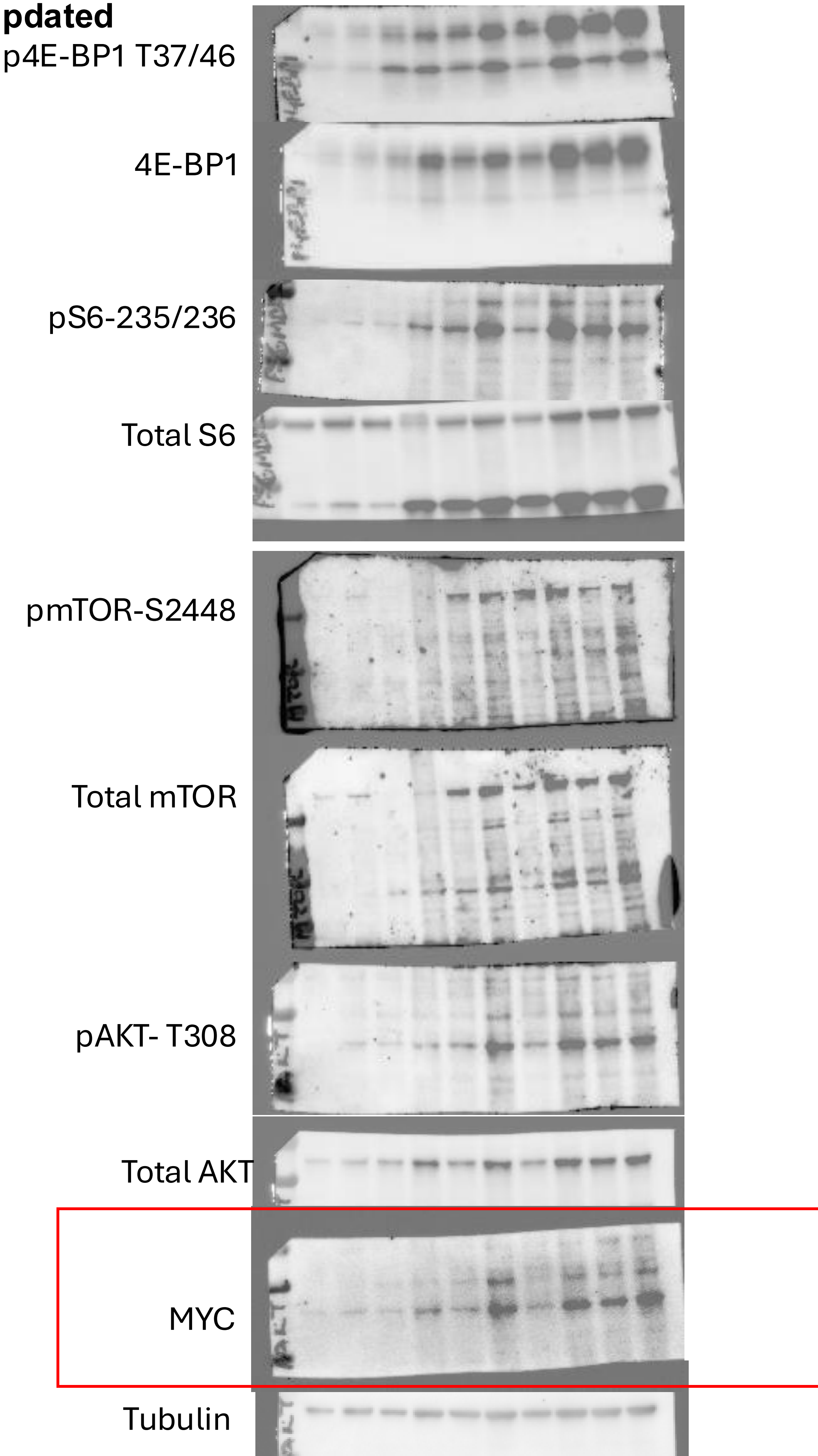

Figure 4G- Moved to Figure 3F

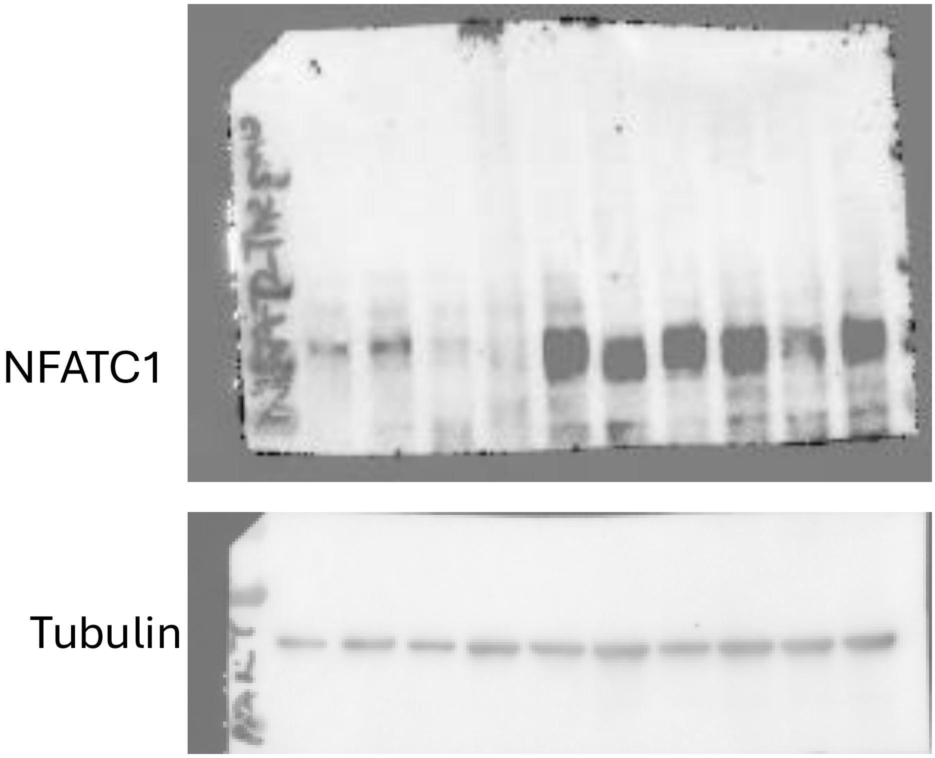

Figure 3F and Figure 4G are same blots done on same day share the same loading control

Figure 4J (Top-right)-

pAKT-T308

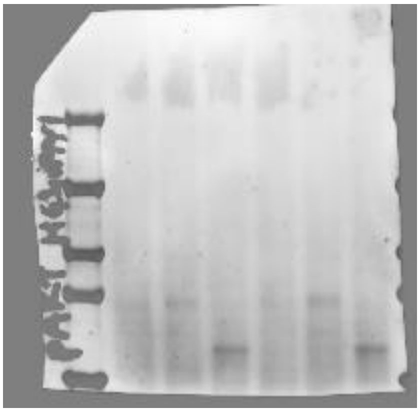

Total AKT

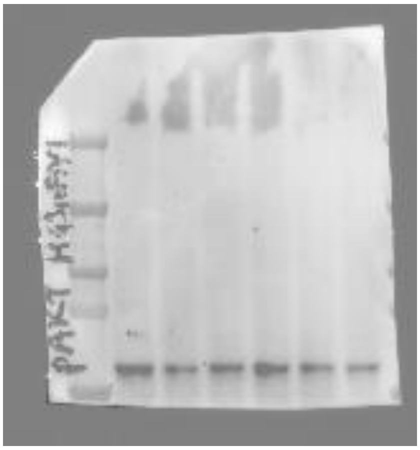

B-tubulin

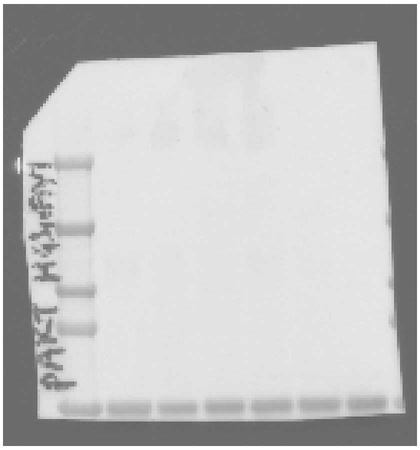

Figure 4J (Bottom-right)-

Phospho -S6 S235/236

Total S6

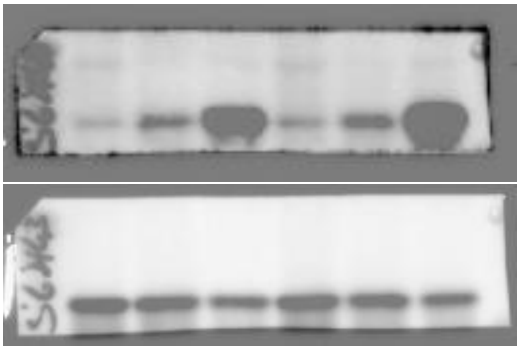

Figure 4J (Top-right)-

NFATC1

p4EBP1-T37/46

Total 4E-BP1

GAPDH

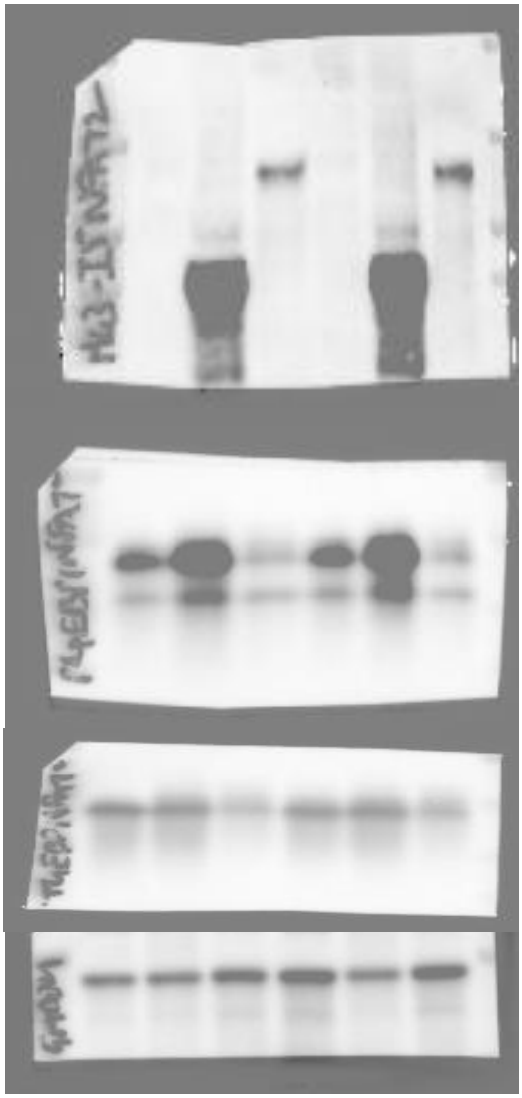

Updated Figure 4J

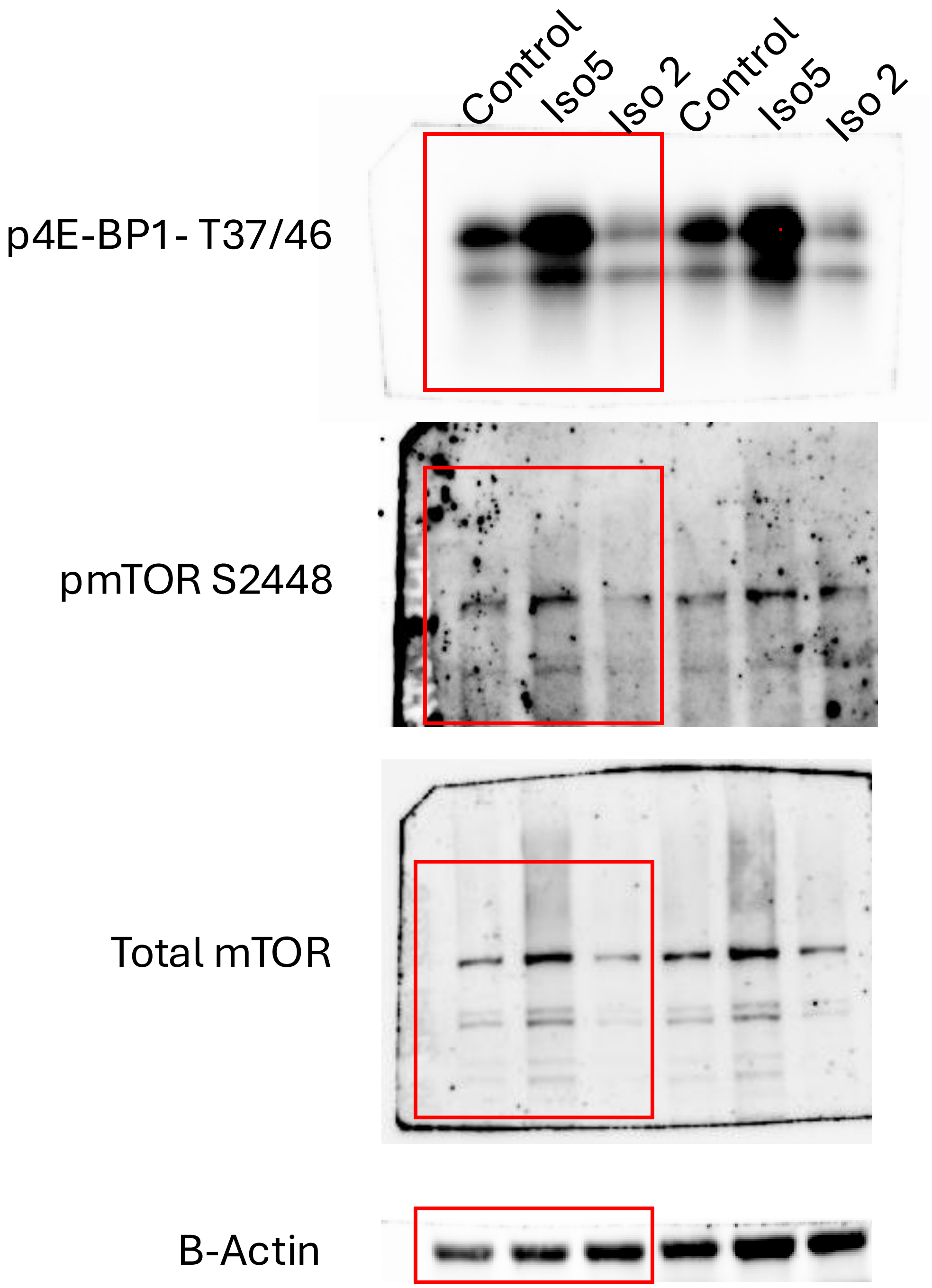

Updated- Figure 4L

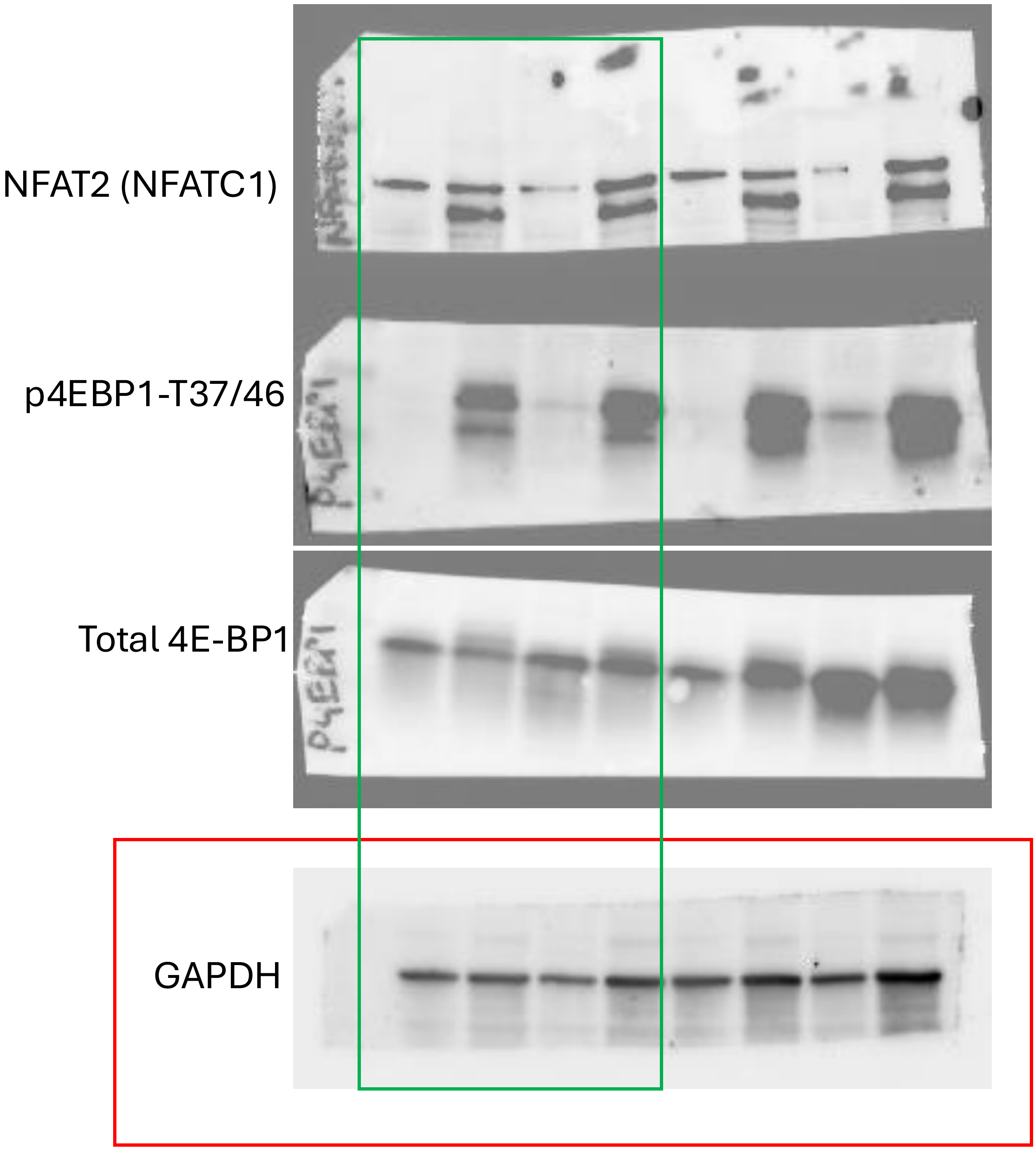

Updated Figure 4L

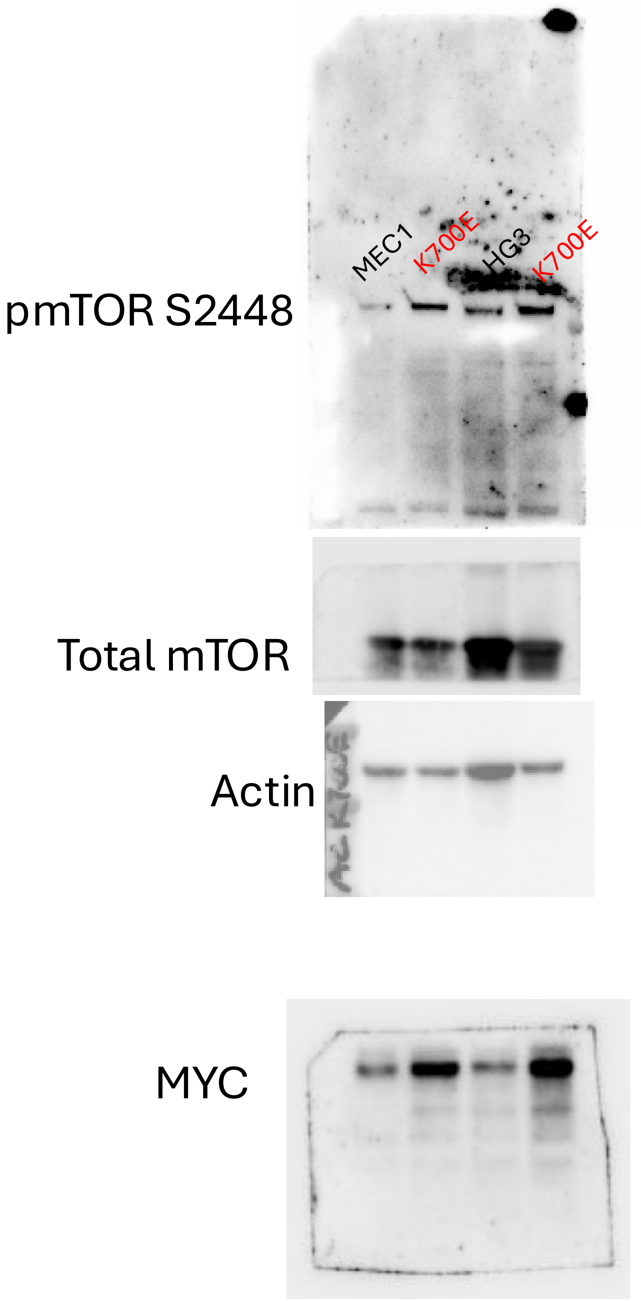

Supplement: Unedited blot and gel images [file jciinsight-10-184280-s032.pdf]
